# Assessing the impact of the addition of pyriproxyfen on the durability of permethrin-treated bed nets in Burkina Faso: a compound-randomized controlled trial

**DOI:** 10.1186/s12936-019-3018-1

**Published:** 2019-12-02

**Authors:** Kobié H. Toé, Frank Mechan, Julie-Anne A. Tangena, Marion Morris, Joanna Solino, Emile F. S. Tchicaya, Alphonse Traoré, Hanafy Ismail, James Maas, Natalie Lissenden, Margaret Pinder, Steve W. Lindsay, Alfred B. Tiono, Hilary Ranson, N’Falé Sagnon

**Affiliations:** 1 Centre National de Recherche et de Formation sur le Paludisme, Ouagadougou, Burkina Faso; 2 Vector Biology Department, Liverpool School of Tropical Medicine, Liverpool, UK; 3 Swiss Centre for Scientific Research in Côte d’Ivoire, Abidjan, Côte d’Ivoire; 4 Department of Biosciences, Durham University, Durham, UK; 5 Medical Research Council Unit, The Gambia at the London School of Hygiene and Tropical Medicine, Banjul, The Gambia

**Keywords:** *Anopheles gambiae*, Burkina Faso, Long-lasting insecticidal nets, Malaria control, Net durability, Olyset, Olyset Duo, Permethrin, Pyriproxyfen

## Abstract

**Background:**

Long-lasting insecticidal nets (LLINs) treated with pyrethroids are the foundation of malaria control in sub-Saharan Africa. Rising pyrethroid resistance in vectors, however, has driven the development of alternative net formulations. Here the durability of polyethylene nets with a novel combination of a pyrethroid, permethrin, and the insect juvenile hormone mimic, pyriproxyfen (PPF), compared to a standard permethrin LLIN, was assessed in rural Burkina Faso.

**Methods:**

A compound-randomized controlled trial was completed in two villages. In one village 326 of the PPF-permethrin nets (Olyset Duo) and 327 standard LLINs (Olyset) were distributed to assess bioefficacy. In a second village, 170 PPF-permethrin nets and 376 LLINs were distributed to assess survivorship. Nets were followed at 6-monthly intervals for 3 years. Bioefficacy was assessed by exposing permethrin-susceptible and resistant *Anopheles gambiae* sensu lato mosquito strains to standard World Health Organization (WHO) cone and tunnel tests with impacts on fertility measured in the resistant strain. Insecticide content was measured using high-performance liquid chromatography. LLIN survivorship was recorded with a questionnaire and assessed by comparing the physical integrity using the proportionate hole index (pHI).

**Results:**

The PPF-permethrin net met WHO bioefficacy criteria (≥ 80% mortality or ≥ 95% knockdown) for the first 18 months, compared to 6 months for the standard LLIN. Mean mosquito mortality for PPF-permethrin nets, across all time points, was 8.6% (CI 2.6–14.6%) higher than the standard LLIN. Fertility rates were reduced after PPF-permethrin net exposure at 1-month post distribution, but not later. Permethrin content of both types of nets remained within the target range of 20 g/kg ± 25% for 242/248 nets tested. The pyriproxyfen content of PPF-permethrin nets declined by 54%, from 10.4 g/kg (CI 10.2–10.6) to 4.7 g/kg (CI 3.5–6.0, p < 0.001) over 36 months. Net survivorship was poor, with only 13% of PPF-permethrin nets and 12% of LLINs still present in the original household after 36 months. There was no difference in the fabric integrity or survivorship between the two net types.

**Conclusion:**

The PPF-permethrin net, Olyset Duo, met or exceeded the performance of the WHO-recommended standard LLIN (Olyset) in the current study but both net types failed the 3-year WHO bioefficacy criteria.

## Background

The massive deployment of long-lasting insecticidal nets (LLINs) across sub-Saharan Africa has been a major factor in the rapid decline of malaria cases in the first 15 years of this century [1]. In Burkina Faso, one of the countries with the highest malaria burden in Africa, a total of 29 million nets have been distributed during three rounds of national LLIN distributions in 2010, 2013 and 2016. Since the start of the net distribution programme malaria-related mortality has declined, yet the drop in number of malaria cases has stalled, with 7.7 million cases in 2015 and 7.9 million cases in 2017 [2]. In rural Burkina Faso, approximately 61% of the population are infected with malaria parasites at any one time [3]. Most LLINs distributed in Burkina Faso to date have been pyrethroid-only nets, with a small number of piperonyl butoxide (PBO) nets distributed in 2010 and 2013 [4]. Resistance to pyrethroid insecticides is widespread in African malaria vectors [5] and has reached exceptionally high levels in Burkina Faso [6]. In the Cascades region of Burkina Faso, the site of the current trial, pyrethroid-only LLINs are no longer effective at killing the local mosquito populations [7].

Efforts to maintain the efficacy of bed nets in areas of pyrethroid resistance have driven the development of alternative net types containing either insecticide synergists, multiple insecticides or insecticides plus insect growth regulators [8]. Data on the efficacy and durability of these ‘next generation’ nets are essential for future control and elimination of malaria. This study compared the bioefficacy and survivorship of novel manufactured nets containing both pyrethroid insecticide and the insect growth regulator pyriproxyfen (PPF) (Olyset Duo nets) with comparator nets containing the pyrethroid alone (Olyset nets). PPF is a World Health Organization (WHO) approved larvicide and is highly effective at inhibiting the emergence of mosquito larvae, although the practical limitations of delivering PPF to the aquatic habitats of anopheline mosquitoes at scale has limited its use in malaria control to date. PPF also interferes with the reproductive output of *Anopheles* spp. and can reduce the longevity of adult mosquitoes [9–11]. Encouraging results from experimental hut studies of Olyset Duo [12–14] led to the first clinical trial of a dual active bed net. A cluster randomized control field trial in an area of intense seasonal malaria transmission and high pyrethroid resistance in the local vector population, found that that the PPF-permethrin net was protective and reduced the clinical incidence of malaria in children by 12% compared to standard pyrethroid-only LLINs [7].

For any candidate LLIN to be recommended by WHO, it requires the demonstration that it is effective under operational conditions for at least 3 years. Following the WHO Pesticide Evaluation Scheme (WHOPES) guidelines, candidate LLINs are assessed on their bioefficacy, insecticide content, and survivorship [15–17]. The current study was undertaken to assess the durability of the PPF-permethrin Olyset Duo net, in the field compared to a standard, WHO approved, permethrin-only net (Olyset). The primary objective was to determine whether the bioefficacy of the PPF-permethrin net was superior to a standard permethrin LLIN over 36 months with secondary and tertiary objectives to compare the physical integrity, net survivorship and insecticide content over the course of the study. This is the first durability study evaluating the performance of ‘next generation’ nets under operational settings.

## Methods

### Study design and study area

A detailed description of the study protocol, which follows WHO guidelines for measuring the durability of LLINs, has been reported previously [18]. In this cluster-randomized controlled trial, the durability of a PPF-permethrin net (Olyset^®^ Duo, 2% permethrin 1% pyriproxyfen, Sumitomo Chemical Ltd) was compared with a conventional pyrethroid LLIN (Olyset^®^, 2% permethrin, Sumitomo Chemical Ltd) incorporated into high-density polyethylene fibres. Nets were ‘Extra Family’ size, rectangular nets (180 cm wide, 90 cm long, 150 cm high, 150 denier) manufactured with an enhanced knitting pattern that was introduced in 2013.

This study enrolled residents of two rural villages, Dalamba (10° 31′ 6.07″ N, 4° 18′ 48.01″ E) and Sanako (10° 36′ 17.56″ N, 4° 22′ 22.17″ E), in Sidéradougou health centre area in Mangodara District, the Cascades region of Burkina Faso. Both villages are approximately 6 km from the study sites of a large clinical trial of these nets [7]. Dalamba comprised of 156 households in 108 compounds and neighbouring Sanako had 111 households in 71 compounds. Compounds are small group of houses typically enclosed by a wall. The houses in the villages are made with mud or cement walls with thatched or metal roofs. At the beginning of this trial, informed consent was provided by the household heads for each household enrolled (Additional file 1). Only one compound head (from Dalamba) refused to participate.

Clustering occurred at compound level with each compound randomly allocated either PPF-permethrin nets or permethrin-only LLINs. A total of 653 nets (326 PPF-permethrin and 327 LLINs) were distributed in Dalamba and 546 nets (170 PPF-permethrin and 376 LLINs) in Sanako from July to August 2014. The distribution of nets was not equal in Sanako as the number of nets was allocated according to the number of people in the compound. The householder and research team were blinded to the net type [18]. During the enrolment and informed consent procedures, householders were encouraged not to exchange the nets. In a separate program, a government-led national LLIN campaign distributed PermaNet 2.0 to all households in the study villages in July 2016, 2 years into the study described here.

In both villages, seven rounds of net sampling and surveys were performed over 36 months from September 2014 until September 2017. Nets were sampled 1 month after distribution and subsequently at 6, 12, 18, 24, 30 and 36 months. In Dalamba, nets were destructively sampled for bioefficacy testing and replacement nets provided. In Sanako, nets were left in situ for assessment of physical integrity and survivorship over the complete length of the trial. An outline of the study design is provided in Additional file 2.

### Bioefficacy (Dalamba village)

Bed nets were sampled from Dalamba according to a pre-defined random sampling schedule [18]. The net number, household identification number, and name of the household head were used by the field team to identify nets for sampling. A total of 48 nets, designed to represent 24 of each net type, were targeted for each collection round but, due to high attrition rates, the total number of nets available to be sampled was consistently lower (Table 1). Three panels of 25 cm^2^ from each net, sampled from three different sides of the net were used in cone bioassays and tunnel bioassays at the Centre National de Recherche et de Formation sur le Paludisme (CNRFP) insectaries in Banfora and Ouagadougou. A further four panels (five in round 1) of 30 cm^2^ were sampled from each net and sent to Liverpool School of Tropical Medicine (LSTM) for additional insecticide content analysis, cone bioassays and fertility analysis.

**Table 1:** Number of nets from Dalamba on which tests were performed in each sampling round

Time after deployment (months)	Chemical content LSTM		Kisumu cone bioassay CNRFP		Tunnel tests CNRFP		Kisumu cone bioassay LSTM		Tiassalé cone bioassay LSTM	
	PPF-permethrin	LLIN	PPF-permethrin	LLIN	PPF-permethrin	LLIN	PPF-permethrin	LLIN	PPF-permethrin	LLIN
1	24	24	24	24	0	0	24	24	24	24
6	22	21	24	21	0	2	22	21	22	21
12	21	23	22	22	1	5	21	23	21	23
18	17	14	17	14	4	7	3	1	17	14
24	15	19	14	20	16	19	14	16	14	16
30	12	12	17	16	12	12	9	3	8	2
36	12	12	20	12	16	10	0	0	0	0

#### Cone tests

Cone tests were performed at the Banfora and Ouagadougou insectaries by CNRFP using batches of five, three to 5-day old unfed *Anopheles gambiae* from the permethrin susceptible Kisumu strain. Ambient conditions in the testing room ranged from 27 ± 2 °C and 80 ± 10% relative humidity. Mosquitoes were exposed to the three 25 cm^2^ panels sampled from each net in triplicates for 3 min. Each net panel was tested four times so that 20 mosquitoes were exposed to each panel, 60 to each net. Mosquitoes were provided a sugar meal post exposure. Knockdown was recorded after 60 min and mortality after 24 h. Four panels 30 cm^2^ from the same nets were sent to LSTM and further cone bioassays performed on three of these panels. For cone bioassays at LSTM both Kisumu and Tiassalé 13 mosquito strains were used; Tiassalé 13 (hereafter Tiassalé) is a pyrethroid resistant strain of *An. gambiae* sensu lato originally from Côte d’Ivoire [19] in which pyrethroid resistance is conferred by a combination of target site and metabolic (cytochrome P450) mechanisms. Tiassalé mosquitoes surviving the cone bioassays were retained for blood feeding and assessed for fecundity as described below.

#### Tunnel tests

Tunnel tests were performed at the CNRFP insectary in Ouagadougou on nets that did not reach the target of ≥ 95% 1 h knockdown or ≥ 80% 24 h mortality after exposure of susceptible mosquitoes in a cone test. Tunnel tests were performed according to WHO protocols [15] using a guinea pig as a host. 100 five to eight-day old unfed female Kisumu mosquitoes were introduced into the tunnel at 18.00 h and the test terminated at 09.00 h the following day. The number of blood fed and dead mosquitoes were counted. A control, using untreated netting, was run for each round. The numbers of nets tested in tunnel assays are shown in Table 1.

#### Reduction in offspring

Nets in Rounds 1–5 were tested for their effect on mosquito reproductive output. At 24 h post exposure, Tiassalé mosquitoes surviving the cone bioassay were offered a human blood meal on an artificial blood feeder (Hemotek, UK) in the dark for 45 min. Mosquitoes exposed to the same net were pooled before blood feeding (maximum of 60 mosquitoes per cup). Unfed mosquitoes were removed, and blood-fed females maintained with access to a sugar solution for a further 3 days. Mosquitoes were then transferred into individual oviposition tubes (flat bottom 30 ml cell culture tubes, Fisher) containing moist cotton wool, covered in filter paper. Tubes were covered with netting and cotton wool soaked in deionized water placed on the netting. Mosquitoes were left for a further 3 days to oviposit. Any filter papers containing eggs were removed and the number of eggs counted under a dissection microscope before floating in approximately 50 ml of water in plastic pots. TetraMin fish food (Tetra, Germany) was added as food for any larvae that hatched. The number of eggs hatched after 2 to 3 days was recorded. Oviposition rate (proportion of survived blood-fed mosquitoes that laid eggs), fecundity (number of eggs laid per survived blood-fed female), hatch rate (proportion of eggs that hatched) and fertility (number of hatched eggs per survived blood-fed female) were compared between mosquitoes exposed to untreated control nets, and mosquitoes exposed to either LLIN or PPF-permethrin nets.

#### Chemical analysis

High-performance liquid chromatography (HPLC) was used to measure the insecticide content of the PPF-permethrin nets and LLINs. Briefly, a representative sample with an area of 48 cm^2^ (approximately 0.2 g net fibre) was cut in triplicates from the four panels of each net (total of 12 samples per net). Net samples were boiled at 85 °C for 45 min with 5 ml solution of 4% 1-propanol in heptane containing 100 μg/ml of internal standard dicyclohexyl phthalate (DCP). 1 ml of the insecticide extract was transferred to a clean glass tube, evaporated to dryness at 40 °C, reconstituted in one ml of acetonitrile and centrifuged at 20,000×*g* for 15 min. HPLC analysis was performed by the injection of 10-μl aliquots of the samples on a reverse-phase Hypersil GOLD C18 column (75 Å, 250 × 4.6 mm, 5-μm particle size; Thermo Scientific) at room temperature. A mobile phase of 70% acetonitrile in water was used at a flow rate of 1 ml min^−1^ to separate permethrin, pyriproxyfen and DCP. Chromatographic peaks of the insecticides and DCP were detected at a wavelength of 232 nm with the Ultimate 3000 UV detector and analysed with Dionex Chromeleon™ 6.8 Chromatography Data System software. Quantities of pyriproxyfen and permethrin were calculated from standard curves established by known concentrations of the insecticide authenticated standards and corrected by internal standard readings in each sample relative to control. Final insecticide content in gram per kilogram (g/kg) net material was estimated using the following equation:

$$I = \left( {\frac{x}{a}} \right) \times \left( {\frac{0.005}{m}} \right) \times C$$

where *I* is the insecticide content in g/kg, *x* is the insecticide peak area at 232 nm obtained from HPLC, *a* is the slope of insecticide standard curve, *m* is the mosquito net sample mass in gram and *C* is the internal standard correction factor calculated from dividing the peak area of 100 μg/ml DCP by the DCP peak area obtained for the unknown. The method accuracy coefficient of variation was lower than 10% for both permethrin and pyriproxyfen extracted from new nets.

### Net survivorship (Sanako village)

In Sanako village, net survivorship was recorded during the seven rounds of data collection. Functional survivorship is defined as the proportion of nets still in households in serviceable condition and is calculated by measuring the total number of nets in ‘good’ + ‘acceptable’ condition (see below) hung over a bed × 100/total number of each net type distributed to surveyed households, excluding the number lost by attrition. At every compound, information on the presence of the distributed nets and their physical condition was recorded and the head of the household or an adult person from the family was asked a set of questions on the use of bed nets in the household (Additional file 3). Distributed nets were identified by a code written with indelible markers on the label of the net. Nets found at a different household than the one where they were originally distributed were excluded from the study.

### Fabric integrity (Sanako village)

Fabric integrity was measured using the results from the questionnaire on the physical condition of the nets. The number of holes, their size and their location on the net was noted. These data were used to calculate the proportion of torn nets, proportion of nets with any holes and the proportionate Hole Index (pHI) as defined by the WHO [16] (pHI = (1 × number of size-1 holes) + (23 × number of size-2 holes) + (196 × number of size-3 holes) + (576 × number of size-4 holes). The pHI ≤ 64 is defined as ‘good’, pHI 65–642 as ‘acceptable’ and pHI ≥ 643 as ‘torn’.

### Data analysis

Data analyses were conducted using the R programming language (version 3.6.0) and plots produced with the ggplot2 package (version 3.2.1). Generalized Linear Mixed Models (GLMMs) (lme4 version 1.1-21) were used to identify factors significantly associated with bioefficacy, chemical analysis, fabric integrity and attrition. To account for unexplained variation between individual nets, the net ID was included in all models as a random effect. The model selection process used stepwise regression with the maximally complex model with all fixed variables and all two-way interactions fit. The contribution of each variable to the explanatory power of the model was evaluated using log-likelihood ratio tests (LRTs). The final model consisted of all explanatory variables which had statistically significant LRTs.

#### Mosquito mortality

Cone test and tunnel test mortality results were adjusted with Abbott’s formula [20], and net types compared over time using GLMMs with a gaussian distribution.

#### Reduction in offspring

The impact of exposure to the different net types on fecundity and fertility was analysed using GLMM.

#### Chemical analysis

The mean concentration, and 95% confidence intervals, of permethrin and pyriproxyfen was calculated and the chemical content contained in the two net types over time was compared using GLMMs with a gaussian distribution.

#### Fabric integrity

The physical integrity of the two net types was compared using: (1) the proportion of torn nets (nets in poor condition), (2) the proportion of nets with holes and (3) the proportionate hole index (pHI) [15, 16]. The following formula were used:

Proportion of torn nets = (Total number of each type of net where the nets are not long enough to be tucked under the mattress, or are torn or badly damaged, or have more than 5 holes (finger width, diameter approx. 2 cm))/(Total number of each net type found and assessed in surveyed households)).

Proportion of nets with any holes = (Total number of nets with any hole/Total number of each net type found and assessed in surveyed households).

#### Net survival

Survivorship of nets was compared using GLMM with a binomial logit link function. The response variable of this model was a binary variable in which a net is either: (1) in functional use at the allocated location or (0) not in functional use and/or not at the allocated location.

### Ethics

The trial was approved by the Ethics Committee for Research in Health, Ministry of Scientific Research and Innovation, Burkina Faso (2014-0-0250) and the School of Biological Sciences ethics committee, Durham University, UK (SBBS/EC/PV120914).

## Results

### Bioefficacy of nets

#### Cone and tunnel tests for pyrethroid susceptible mosquitoes

**Fig. 1:**
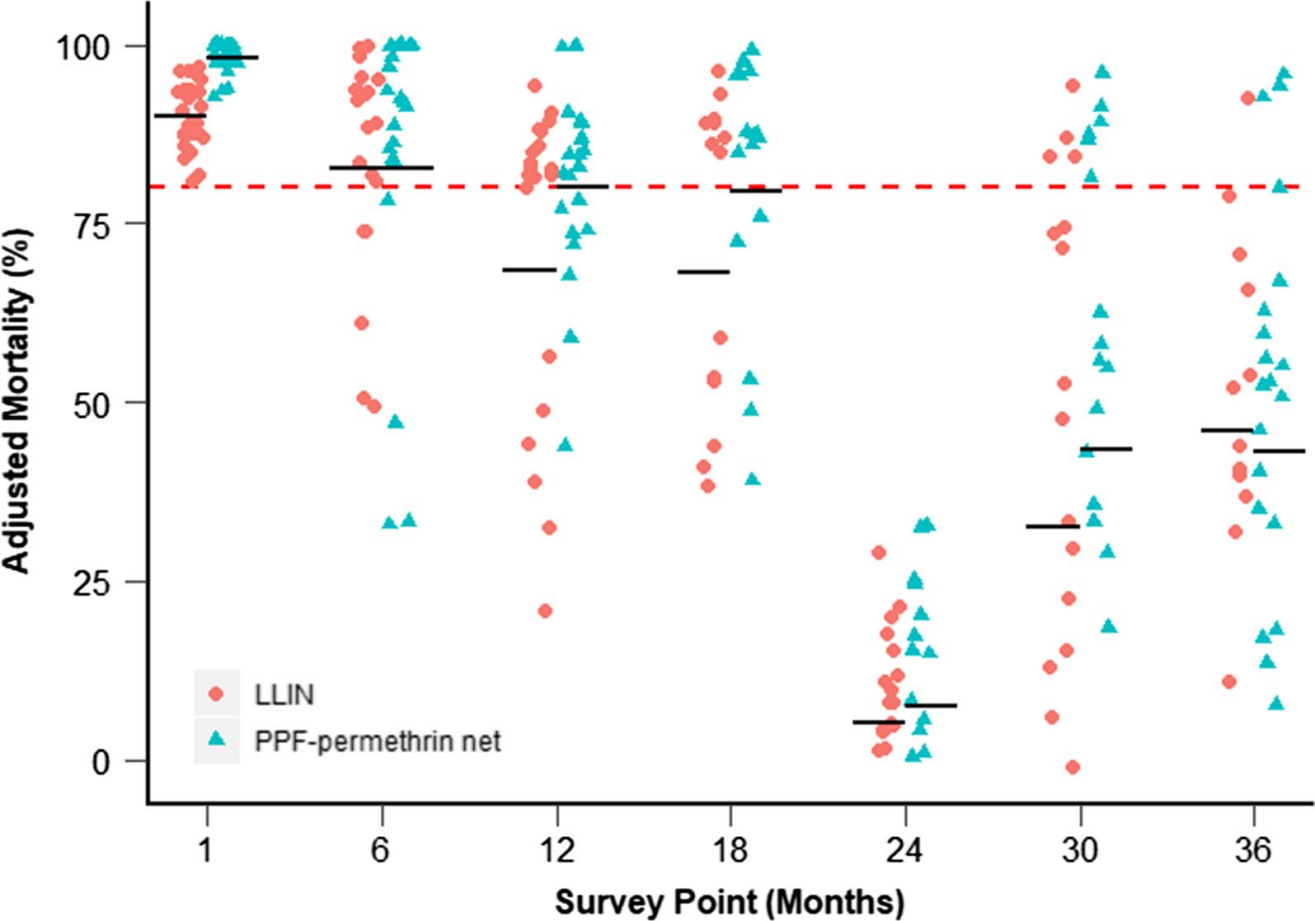
Adjusted mortality of susceptible *Anopheles gambiae* (Kisumu strain) mosquitoes exposed in cone bioassays to PPF-permethrin nets and LLINs at CNRFP. Horizontal black bars indicate geometric mean mortality. Horizontal red dotted line indicates 80% mortality threshold

A total of 138 PPF-permethrin nets and 128 LLINs were tested with WHO cone bioassays on pyrethroid susceptible *An. gambiae* sensu stricto (s.s.) (Kisumu strain) in Burkina Faso at CNRFP (Fig. 1). Additionally, a proportion of these nets (84 PPF-permethrin nets and 85 LLINs) were also tested on the Kisumu strain at LSTM for validation of results (Additional file 4).

Mean mortality for the permethrin-only LLIN met the 80% threshold at baseline and 6 months but fell below this threshold from 12 months onwards; the threshold mortality for the PPF-permethrin nets was reached for the first 18 months. Dramatically reduced levels of mortality were observed for both net types at the 24-month sampling point (PPF-permethrin 10.0% and LLIN 6.9%). Mortality, however, increased unexpectedly again at 30 and 36 months. A similar pattern in efficacy was observed for the susceptible Kisumu strain at LSTM (Additional file 4), with mortality for both net types at its lowest at 24 months and increasing again at 30 months (the final nets from 36 months were not evaluated at LSTM). Knockdown data showed trends similar to the mortality data, with very low knock down rates at 24 months and higher rates in subsequent months (Additional file 5).

The best fit model determined that the mean mortality for PPF-permethrin nets over time was 8.6% (CI 2.6%–14.6%) higher than for the LLINs (*χ*^2^ = 11.244, df = 1, p < 0.006). Comparison with the null model indicated that the model explained approximately 58.6% of total deviance in the cone mortality data.

**Fig. 2:**
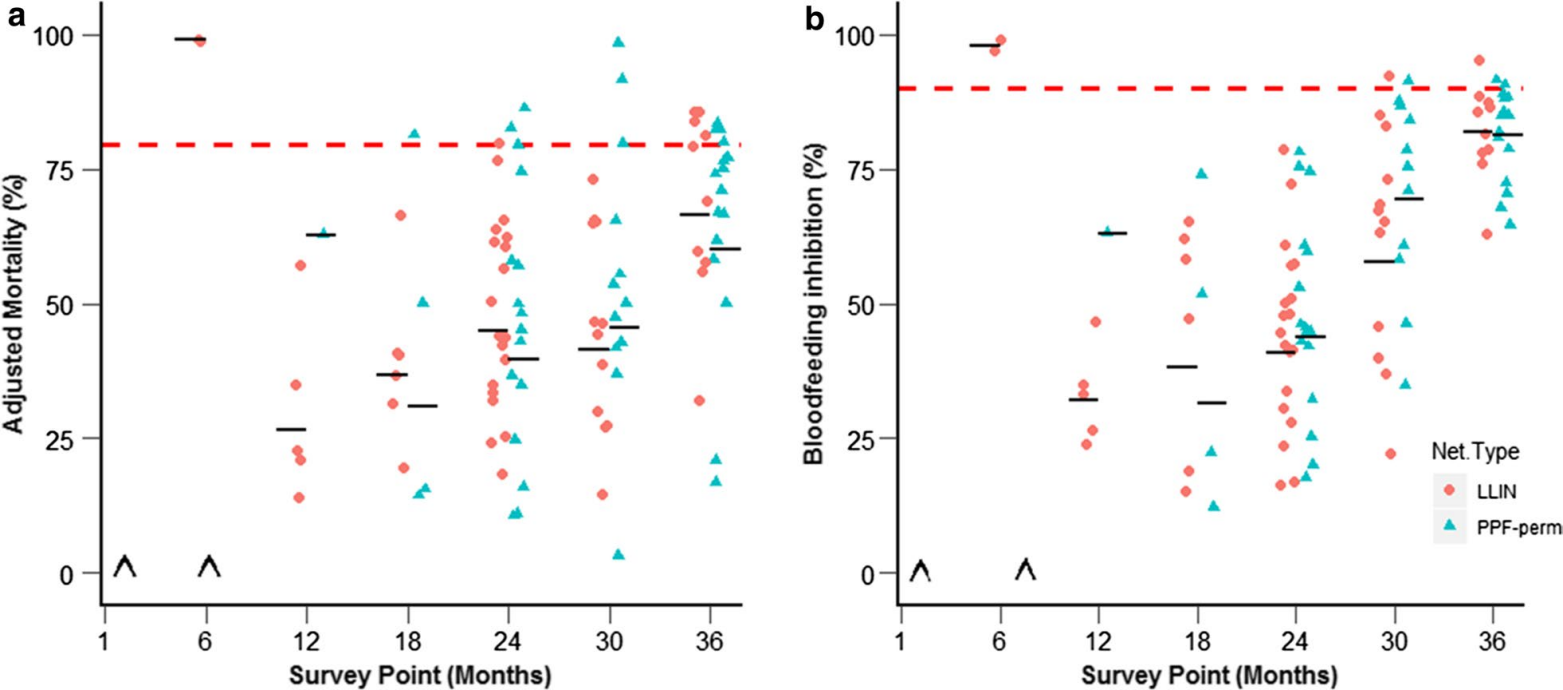
Bioefficacy of pyrethroid-susceptible *An. gambiae* s.s. (Kisumu strain) mosquitoes exposed in tunnel tests to each net type. **a** Adjusted mortality and **b** blood feeding inhibition. Horizontal red dotted lines indicate thresholds (≥ 80% for mortality and ≥ 90% for blood feeding inhibition). ^∧^Indicates survey points when no tunnel tests were conducted

Tunnel tests (Fig. 2), performed on the 104 (49 PPF-Permethrin and 55 LLINs) nets which did not meet the WHO criteria of ≥ 95% knockdown and/or ≥ 80% mortality after cone tests, showed that 19 nets passed the tunnel test criteria for mortality and/or blood feeding, of which 11 were PPF-permethrin nets and 9 LLINs (Table 2).

**Table 2:** Summary of cone and tunnel test results

	Net type	Time after net distribution (months)						
		1	6	12	18	24	30	36
Cone test	PPF-permethrin	100% (24/24)	100% (23/23)	95.4% (21/22)	76.47% (13/17)	0% (0/14)	37.5% (6/16)	20.0%(4/20)
	LLIN	100% (24/24)	90.0% (18/20)	72.7% (16/22)	57.14% (8/14)	0% (0/20)	25.0% (4/16)	16.6% (2/12)
Tunnel test	PPF-permethrin	N/A	N/A	0% (0/1)	25% (1/4)	21.4% (3/14)	30.0% (3/10)	25% (4/16)
	LLIN	N/A	100% (2/2)	0% (0/5)	0% (0/6)	5.55% (1/18)	0% (1/12)	50% (5/10)
Overall^a^	PPF-permethrin	*100%* (*24/24*)	*100%* (*23/23*)	*95.4%* (*21/22*)	*82.35%* (*14/17*)	***21.4% (3/14)***	***56.25%*** (***9/16***)	***66.66%*** (***8/12***)
	LLIN	*100%* (*24/24*)	*100%* (*20/20*)	***72.7%*** (***16/22***)	***64.3%*** (***8/14***)	***5%*** (***1/20***)	***31.25% (5/16)***	***58.33% (7/12)***

WHO cut off criteria for cone bioassay is ≥ 95% knock down and/or ≥ 80% mortality. For tunnel tests cut off criteria is ≥ 80% mortality and/or ≥ 90% blood feeding inhibition

Italic indicates pass, bolditalic indicates fail

*N/A* No nets tested

^a^Nets meet overall WHO criteria for a given timepoint if 80% of nets pass either cone or tunnel tests

Combining the results from the cone bioassays and the tunnel tests, the PPF-permethrin net met the WHOPES bioefficacy criteria for 12 months longer than the standard permethrin-only LLIN (18 months compared to 6). At the final time point of 36 months post distribution, just 67% of PPF-permethrin nets (8/12) and 58% of standard LLINs (7/12) met the WHOPES bioefficacy criteria (Table 2).

#### Cone tests for pyrethroid resistant mosquitoes

**Fig. 3:**
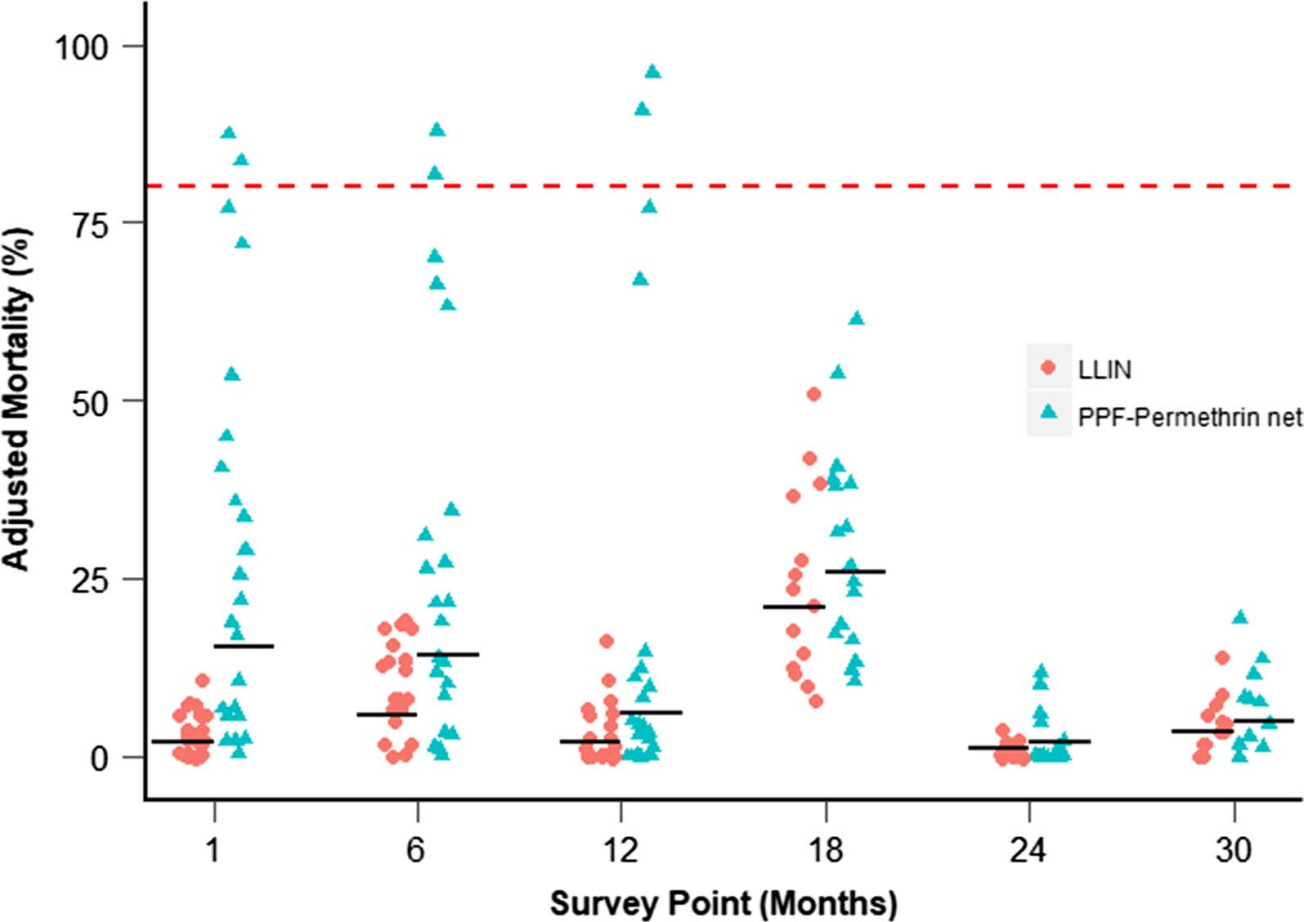
Adjusted mortality of resistant *An. gambiae* (Tiassalé strain) mosquitoes exposed in cone bioassays to LLINs and PPF-permethrin nets at LSTM. Horizontal black bars indicate geometric mean mortality. Horizontal red dotted line indicates 80% mortality threshold

Pyrethroid-resistant *An. gambiae* s.s. (Tiassalé strain) were used to test the bioefficacy of 106 PPF-permethrin nets and 100 LLINs at LSTM using WHO cone bioassays. Mosquito mortality over all time points was 14.6% (CI 9.7–19.5%) higher for PPF-permethrin nets than LLINs (*χ*^2^ = 43.55, df = 2, p < 0.001, Fig. 3). Mortality, however, was markedly more variable when mosquitoes were exposed to PPF-permethrin nets than LLINs. At 18 months and after, there was no difference in mortality between net types (18 months: p = 0.42, 24 months: p = 0.74, 30 months: p = 0.72). Comparison with the null model indicated that the model explained approximately 58.6% of total deviance in the data. Knockdown data are shown in Additional file 6.

#### Reduction in offspring

Pyrethroid-resistant mosquitoes that survived exposure in cone bioassays, were offered a blood meal and then retained for oviposition assays. Of 10,793 pyrethroid-resistant mosquitoes exposed to either net type, 56.3% (6078) survived 24 h after blood feeding and 3018 (49.7%) of the survivors laid eggs. Oviposition rates in mosquitoes exposed to untreated nets were variable, ranging from 0.43 to 0.96 in the different survey periods (Additional file 7). No comparisons were made for nets 18 months post-distribution as only one successful control exposure was done.

**Fig. 4:**
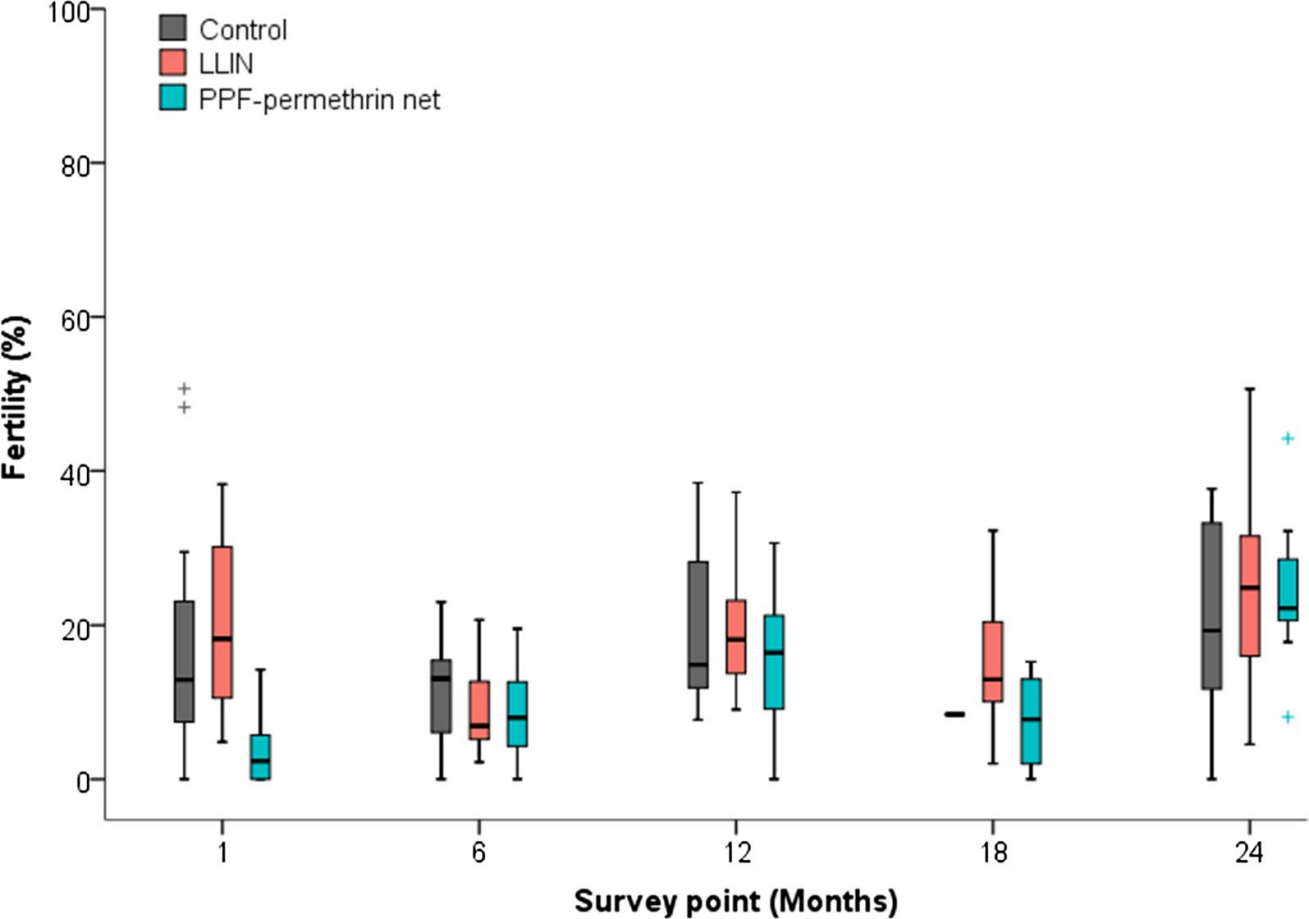
Fertility of resistant *An. gambiae* (Tiassalé strain) mosquitoes after exposure to LLINs and PPF-permethrin nets. The fertility rate is the number of larvae per survived blood-fed female for a given treatment

One-month post-distribution fertility was 80% lower for mosquitoes exposed to PPF-permethrin nets compared to mosquitoes exposed to the control nets (p = 0.0001), while there was no difference between LLINs and controls (p = 0.439, Fig. 4). The reduction in fertility in PPF-permethrin nets was due to 40% fewer mosquitoes laying eggs (oviposition p = 0.01), which resulted in 47% fewer eggs laid (fecundity p = 0.007). Additionally, the eggs had a 60% lower hatch rate (p < 0.001) than eggs laid by control mosquitoes (Additional file 7). There was no significant impact on fertility of the PPF-permethrin nets beyond 1 month (Fig. 4 and Additional file 7).

#### Chemical analysis

**Fig. 5:**
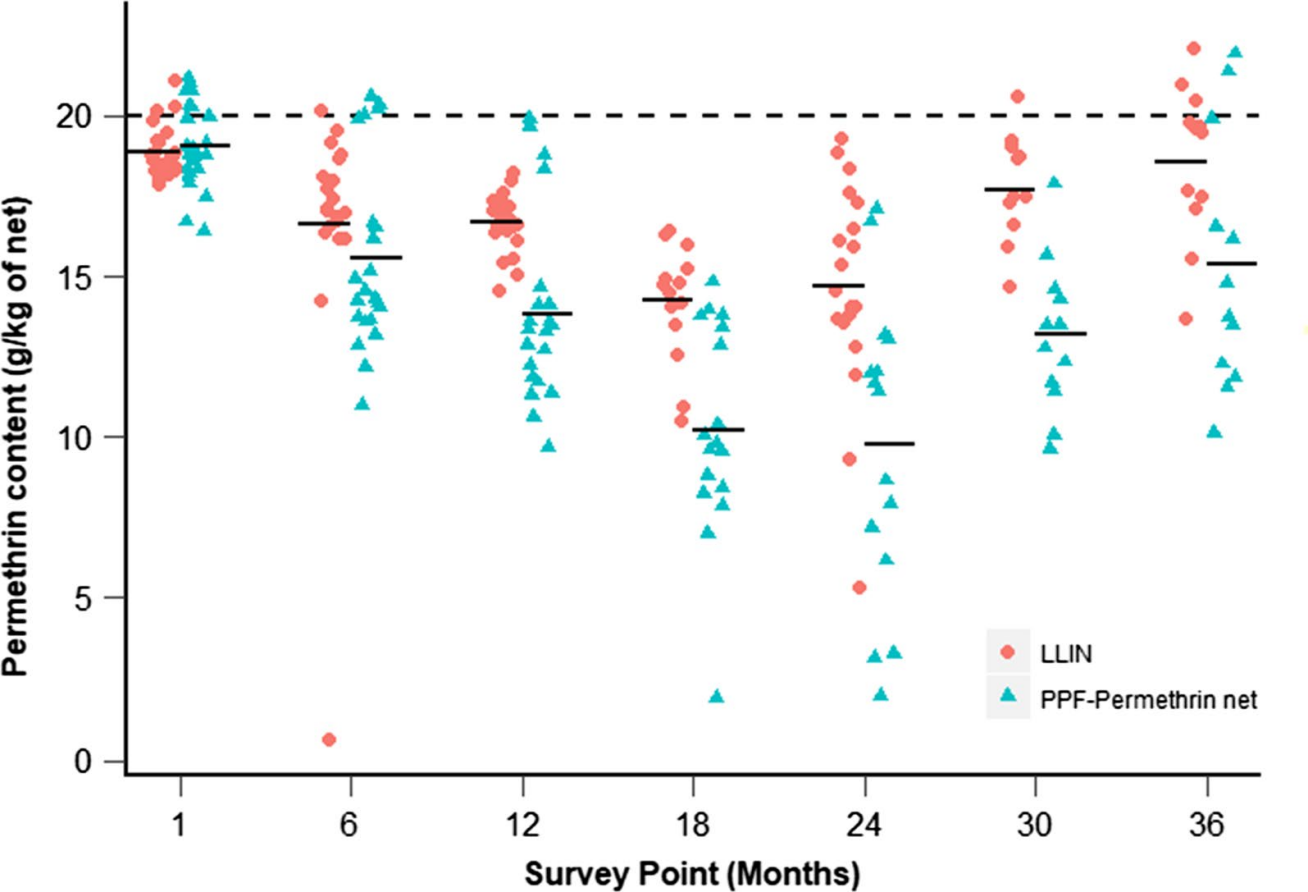
Permethrin content (g/kg netting) for LLINs and PPF-permethrin nets across the sampled time points. Horizontal black bars indicate geometric mean permethrin content. The horizontal black dotted line at 20 g/kg indicates the manufacturers target dose of permethrin for new nets

The insecticide content of 123 PPF-permethrin nets and 125 LLINs was measured. The permethrin content of both net types declined over 18 months and then rose from 24 months onwards (*χ*^2^ = 505.33, df = 1, p < 0.001, Fig. 5). Permethrin content of PPF-permethrin nets fell from 19.1 g/kg (95% CI 18.5–19.6) to 10.20 g/kg (95% CI 8.7–11.8) at 18 months and rose back to 15.4 g/kg (95% CI 13.1–17.6) at 36 months. Permethrin content of the LLINs fell from 18.9 g/kg (95% CI 18.6–19.4) to 14.3 g/kg (95% CI 13.3–15.2) and rose back to 18.6 g/kg (95% CI 17.2–19.9) at 36 months. At the end of the study, at 36 months, the permethrin content of LLINs was similar to nets collected immediately after distribution (p = 0.99).

The permethrin content of the two net types differed across the sampled time points (Fig. 5), with the permethrin content in PPF-permethrin nets 14% (2.59 g/kg, 95% CI 1.86–3.31) lower than that of LLINs (*χ*^2^ = 44.067, df = 1, p < 0.001). Comparison with the null model indicated that the best-fit permethrin model explained approximately 42.5% of total deviance in the data.

**Fig. 6:**
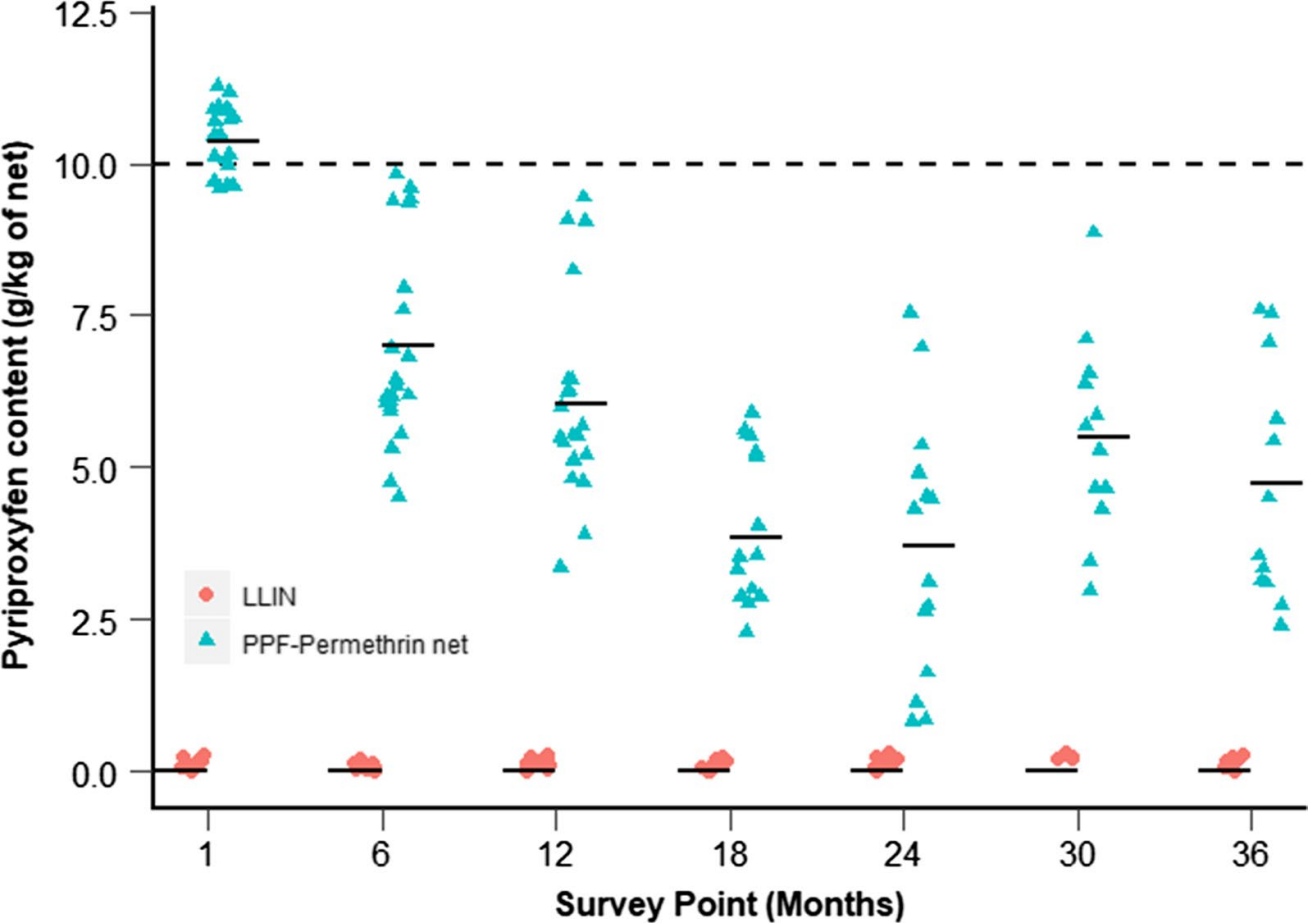
Pyriproxyfen content (g/kg netting) for LLINs and PPF-permethrin nets across the sampled time points. Horizontal black bars indicate geometric mean pyriproxyfen content. The horizontal black dotted line at 10 g/kg indicates the manufacturers target dose of pyriproxyfen for new nets

The pyriproxyfen content of PPF-permethrin nets declined for the first 24 months of the study but again, a small increase in the concentration of PPF was observed for nets surveyed in year three (Fig. 6). From immediately post distribution to 36 months, the mean pyriproxyfen concentration in permethrin-PPF nets had declined by 54% from 10.4 g/kg (CI 10.2–10.6) to 4.7 g/kg (CI 3.5–6.0, GLMM, p < 0.001).

### Fabric integrity

**Fig. 7:**
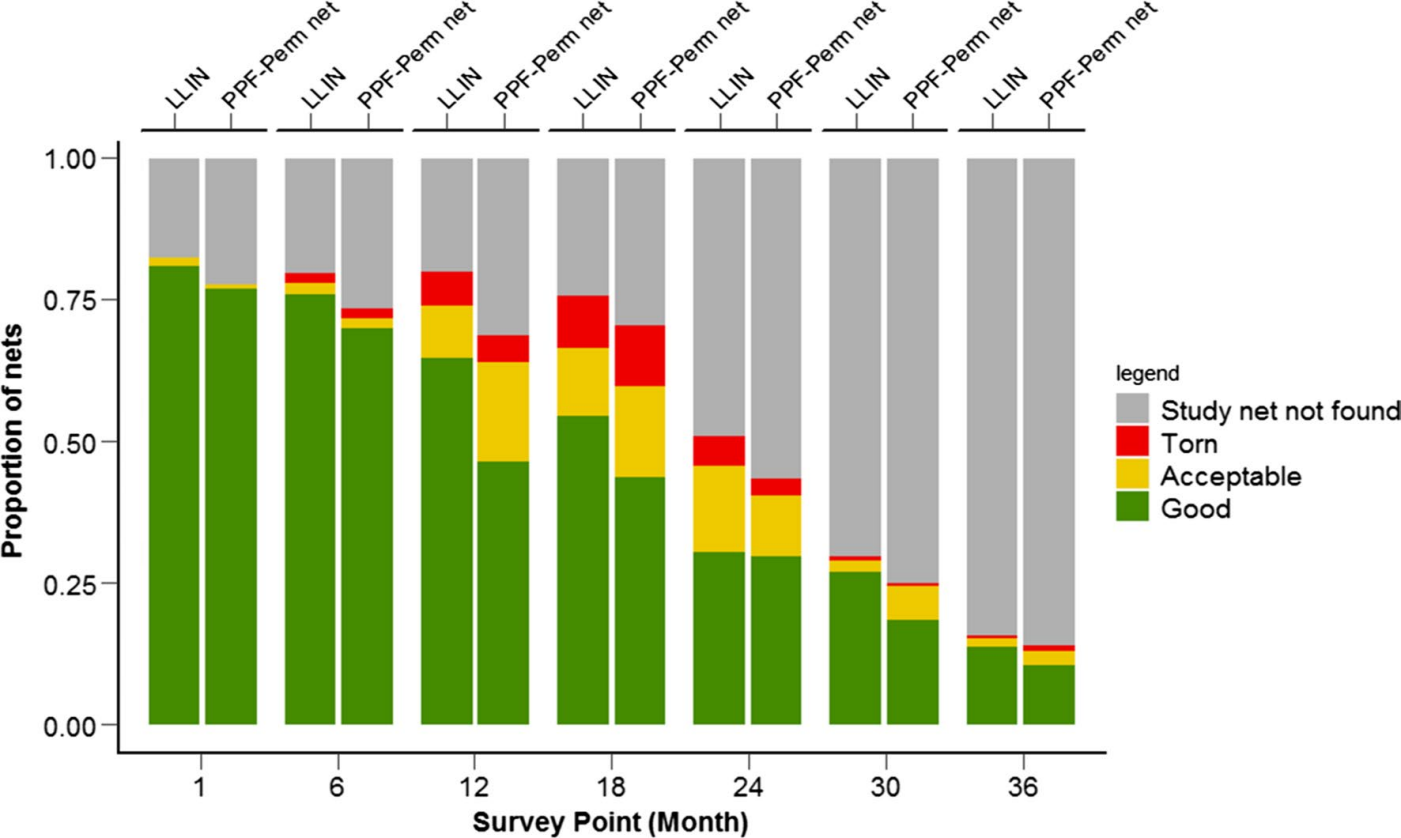
Proportion of PPF-permethrin nets and LLINs in each pHI category (and those not found) across the seven time points of the survey

The proportion of torn nets was low throughout the study for both net types, ranging from 0% at 1 month to its peak of 11% at 18 months post-distribution. There was no difference in the proportion of torn nets between net types (p = 0.089). However, the odds of a net having at least one hole was 47% higher overall for PPF-permethrin nets LLINs (*χ*^2^ = 15.77, df = 1, p < 0.001). The proportion of project nets in ‘good’ condition decreased during the study, falling from 77.1% (131/170) at 1 month to 10.6% (18/169) at 36 months for PPF-permethrin nets and 81.1% (304/375) to 13.9% (52/374) for LLINs. This is largely driven by net attrition with the majority of nets lost from households after 24 months (Fig. 7).

At 36 months, when lost/discarded nets are excluded, nets in ‘good’ condition accounted for 75% (18/24) and 88.1% (52/59) of remaining PPF-permethrin nets and LLINs respectively.

### Net survival

There was a decline in functional survivorship during the study, with only 13.1% of PPF-permethrin nets and 12.4% of LLINs found hanging in the compound they were originally distributed to, in acceptable or good condition after 3 years. There was no difference between the survivorship of PPF-permethrin nets and LLINs at any point in the study (*χ*^2^ = 0.126, df = 1, p = 0.72). Comparison with the null model indicated that the best fit model explained approximately 36.4% of total deviance in the data.

**Fig. 8:**
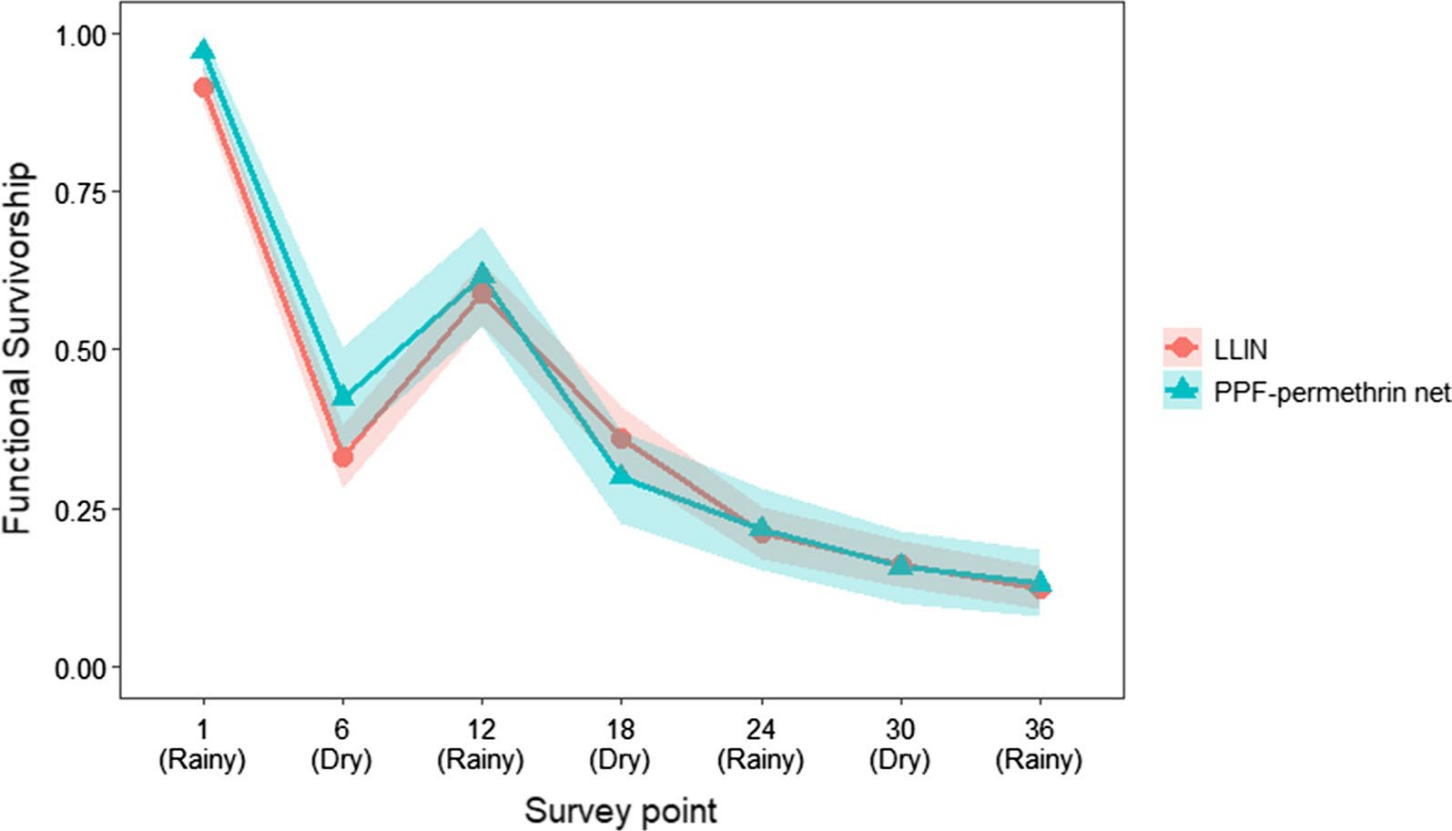
Mean functional survivorship of PPF-permethrin nets and LLINs. Nets that were unused at all time points in the study were excluded. (Rainy) for rainy season, (Dry) for dry season

The overall change in functional survivorship across the study was non-linear with a distinct second peak at 12 months post-distribution (Fig. 8). The model attributed this non-linearity to seasonal variation, with the odds that a net was in use at its designated location increasing during the rainy season (Table 3). The positive effect of the rainy season on survivorship declined in magnitude as the study progressed.

**Table 3:** Odds Ratios and 95% confidence intervals for impact of modelled variables on net survivorship

Variable	Odds ratio	95% confidence intervals	p-value
Net type			
(LLIN)	1.00		
PPF-permethrin net	1.07	0.73–1.56	0.72
Survey No.			
1	0.74	0.68–0.80	< 0.001*
Season			
(Dry)	1.00		
Rainy	24.50	15.79–38.02	< 0.001*
Survey No.: season (rainy)	0.55	0.52–0.61	< 0.001*

* Significant impact on functional survivorship, p < 0.05

### Sampling bias

**Fig. 9:**
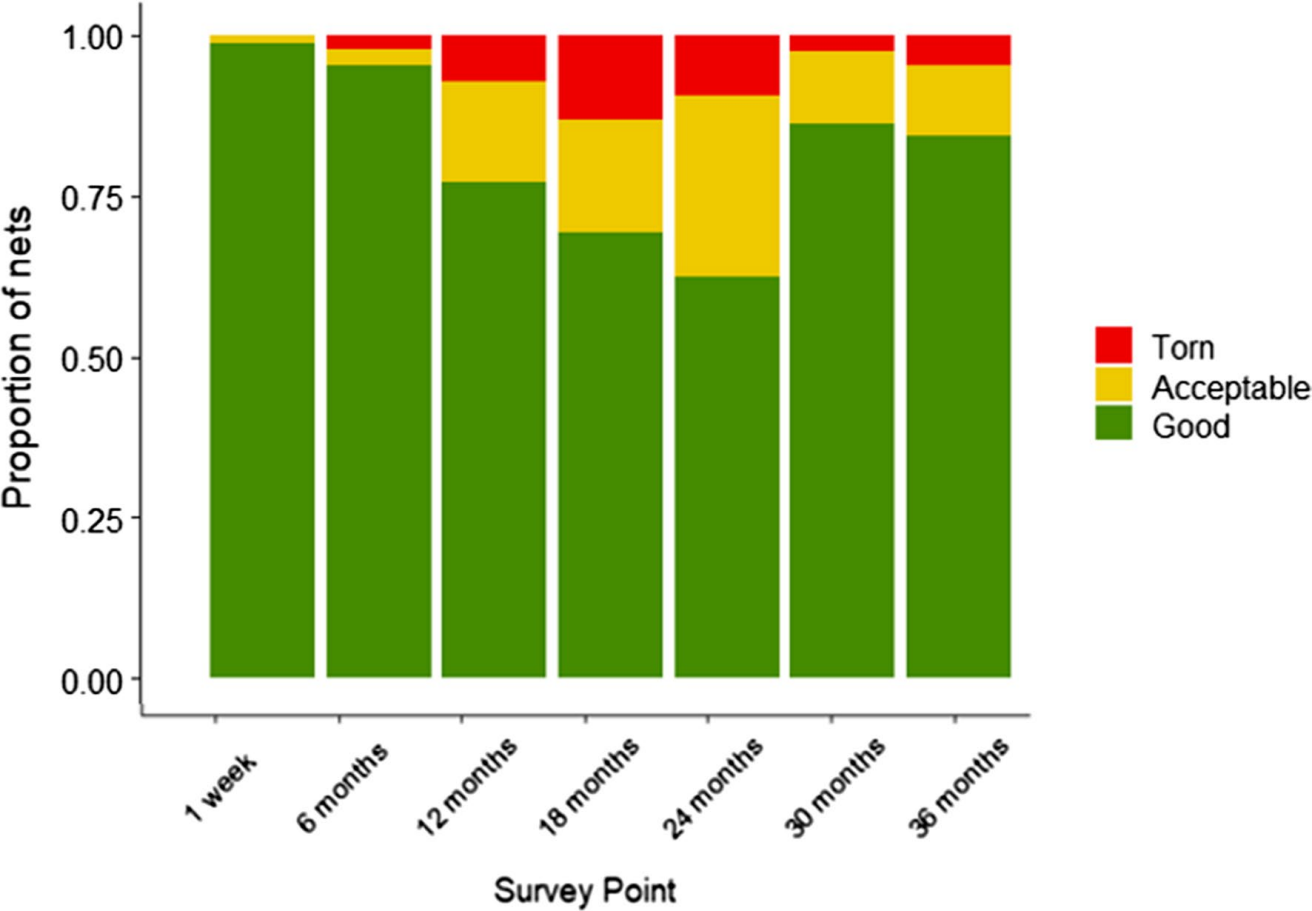
The proportion of PPF-permethrin nets and LLINs (excluding missing nets) in each pHI category across the seven time points of the survey

The unexpected changes in the chemical content and associated bioefficacy observed in this study are indicative of a sampling bias in the quality of nets collected at each timepoint. As measurement can only be performed on nets that remain to be sampled, those nets that are destroyed or lost are censored from sampling. If nets in poor physical condition are disproportionately more likely to be discarded over time than nets in good condition, random samples of the remaining nets will be biased towards higher quality nets. This bias towards nets in better conditions in later time points can be directly observed when considering the physical integrity of remaining nets at each time point (Fig. 9).

## Discussion

### Bioefficacy of nets

This study was the first to measure the durability of dual active ‘next generation’ nets under operational conditions according to the WHOPES criteria on bioefficacy, insecticide content, and survivorship. Both net types met the bioefficacy criteria for 6 months post distribution, however, the standard Olyset LLIN failed thereafter. The candidate PPF-permethrin net, Olyset Duo, continued to meet the threshold for a further 12 months than the LLIN, remaining effective until 18 months post-distribution. Typically, nets are only recommended by the WHO provided they meet the defined bioefficacy criteria for 3 years however older LLIN products on the market may have instead been evaluated under less-stringent guidelines that are no longer in use. Importantly, the standard LLIN used in this comparison, did not fulfil the criteria needed for approval; a similar observation was made in a recent study in Tanzania [21]. Contrary to these results, a retrospective study in Tanzania carried out in 2013 found that more than 96% of Olyset nets in serviceable condition passed the WHO cone/tunnel tests cut off criteria 36 months post distribution [22]. Similarly, a study in western Kenya from 2015 measured 100% mortality up to 3 years post-distribution for Olyset nets [23]. Both East African surveys were based on one sampling point, and, as the results in this study show, it is difficult to interpret net durability data in the absence of intermediate data on net usage over time. Olyset, the standard LLIN used as the comparator in this study, has been distributed to millions of people in sub-Saharan Africa, so the underperformance of this net is a cause of great concern. At a time when malaria prevalence is rising in several sub-Saharan African countries [2] it is important that the bioefficacy of Olyset and other types of nets are monitored when deployed to ensure that they provide protection for at least 3 years.

Tests with pyrethroid-resistant mosquitoes showed that the PPF-permethrin net provided greater bioefficacy for the first 18 months than did standard LLINs but declined to very low levels in both nets thereafter. The increased mortality found with Olyset Duo nets could be attributed to the higher permethrin bleed rate compared to Olyset, as has been previously proposed [12, 13]. Although the results of the chemical analysis found that the mean permethrin concentration was lower in PPF-treated nets than LLINs, the concentration of permethrin detected in net fibres is not necessarily representative of the bioavailability of permethrin to a mosquito on the surface. Alternatively, or in addition, as both pyrethroids and pyriproxyfen have been shown to be metabolized by the same mosquito cytochrome P450s, this increased mortality observed in the PPF-permethrin combination may be indicative of a saturation effect whereby mosquitoes are unable to simultaneously detoxify the two active ingredients on the net [24]. These findings are supported by those from experimental hut trials in Benin and Côte d’Ivoire, where *Anopheles* are highly resistant to pyrethroids, also found higher levels of mortality with PPF-permethrin nets than LLINs [12, 13].

The sterilizing effect of the PPF-permethrin nets was short lived. One-month post distribution the fertility of mosquitoes exposed to PPF-permethrin nets was 80% lower than mosquitoes exposed to either untreated or standard LLINs but no significant reduction in fertility was observed at any subsequent time point. This contrasts with entomological data collected during a clinical trial [7] which found evidence of a strong sterilizing effect of PPF-permethrin nets 1-year post distribution (Grisales, manuscript in preparation). Both laboratory cone bioassays and experimental hut studies on PPF-permethrin nets found that net washing reduced the sterilizing effect of PPF but the impact of washing was much greater in the cone bioassays [14]; the authors attribute this to the short duration of the cone bioassay which may not allow mosquitoes to pick up sufficient PPF to cause complete sterilization. However, this explanation is not supported by video tracking studies which show that the actual time mosquitoes spend in contact with a LLIN is very low, and typically less than 3 min [25]. Nevertheless, it would be informative to perform tunnel tests, or similar tests with longer exposure times on the pyrethroid resistant strain to assess the duration of the sterilizing effect of the PPF in the Olyset Duo nets under controlled conditions.

### Net survivorship

The secondary objective of this study was to determine the physical integrity of the two net types for the first 36 months after distribution. There was little evidence of a difference in the physical durability of the two nets. A higher proportion of the PPF-permethrin nets had at least one hole but neither the proportion of torn nets nor the proportional hole index differed between the two net types. The attrition rate observed for both net types was very high with only 12% of all PPF-permethrin nets and 13% of standard permethrin only LLINs in good or acceptable condition *and* present in the household to which they were originally assigned 36 months post-distribution. The similar rates of survivorship between the two net types indicates that the high attrition is associated with the environmental or sociological characteristics of the setting rather than any differential preference or physical durability of either net type. Unfortunately, although the survey data did ask follow-up questions if the net was not found in the expected location, the large amount of missing data and/or ambiguous answers precluded us from accurately assigning causes for net attrition. A meta-analysis of data from 14 different national net distribution campaigns found that a similar proportion of nets that had left the household had been given away, rather than destroyed [26]. The national distribution of PermaNet 2.0 LLINs to the study site approximately 24 months into the trial may have contributed to the accelerated attrition of study nets. When individuals received these new government distributed nets, they may have then had less incentive to utilize, maintain and/or retain their existing study net, as has been described previously in qualitative surveys conducted on LLIN users in Senegal [27].

This study explicitly quantifies the impact of seasonal changes on the functional survivorship of the candidate LLIN. The arrival of the rainy season had a strong positive effect on the probability that a net would be hanging up in a sleeping place (taken to be an indicator of use). It is expected that an individual’s motivation to use their net would be increased by the higher biting densities of mosquitoes associated with the rainy season. Additionally, this could also be interpreted as the dry season having a strong negative effect on the probability that a net being used, due to the discomfort of sleeping under a net during hot nights. This fluctuation in functional survivorship over time highlights that net attrition is not necessarily permanent and nets may return to functional use at a later time point. Future evaluations of novel LLINs should consider the impact of seasonal variation on net utilization when attempting to model survivorship over time.

### Survivorship bias

Observations of the bioefficacy and chemical analysis data highlight a seemingly counterintuitive finding. The performance of the nets decreases for the first 2 years of use and then increases. As the sampling process is destructive, the same nets are not sampled at each time point. Nevertheless, it is initially difficult to explain why insecticide content and bioefficacy could increase over time. It is possible that there is a survivorship bias in the sampling methodology. If nets in poor physical condition are disproportionately more likely to be discarded over time than nets in good condition, random samples of the remaining nets will be biased towards nets in good condition. This is consistent with a survivorship bias where an unrepresentative subgroup of nets disproportionately persisted to the latter stages of the trial, accounting for an increasingly large proportion of the remaining nets. This bias towards nets in better conditions in later time points can be directly observed when considering the physical integrity of remaining nets at each time point. This illustrates that the proportion of nets in ‘torn’ condition is not a reliable metric of durability in itself as it may be biased by non-random discarding of nets as the study progresses.

The apparent increase in net quality at 30- and 36-months post-distribution (as evidenced by chemical analysis and bioassays on the susceptible strain) could also be accounted for by users not utilizing their assigned nets immediately and these nets then being introduced at later time points. Even 1 month after net distribution, only three quarters of the nets were in use in the expected households. It is possible that some of the survey nets were only utilized after other nets in the household became too torn, or as household number increased. This could potentially explain the phenomenon in the chemical and biological efficacy data where performance of both net types was lowest at 24 months and then increased again at 30 and 36 months. Unfortunately, it was not possible to reliably ascertain whether nets were detected in use for the first-time mid-way through the study.

### Potential improvements to study protocol

The study protocol was based on WHO recommendations for evaluating LLINs in the field [15, 16], but for combination LLINs, like PPF-pyrethroid nets, there are a number of ways in which the protocols could be improved.

1. Cone bioassays may not be the most appropriate method for assessing bioefficacy for all active ingredients, particularly those with repellent or contact irritant properties where contact with the net may be less than the targeted 3 min. Poor results from cone bioassays on Olyset nets have been reported by us and others previously [28, 29]. Nevertheless, in the current study, nets that failed cone tests generally also performed poorly in tunnel tests, suggesting that the duration of contact was not responsible for the low mortality rates.
2. Bioassays to assess the impact of the nets on mosquito fertility were extremely time consuming to complete. The results were also confounded by low levels of oviposition in the controls (typically less than 50%). This has also confounded a previous study [13]. An alternative and less labour-intensive method to assess the impact of pyriproxyfen exposure is to examine the morphology of the ovaries. However, further work is required to determine the correlation between these two measurements.
3. In general, to assess the bioefficacy of dual active ingredient nets, it is necessary to use a strain with resistance to one of the two insecticides. It is important that the same strain is used throughout, and that resistance is maintained at a stable level throughout the study. This is challenging when studies last for 3 years. An alternative option might be to test all of the net samples from the different collection rounds at the end of the study but further information about the stability of the active ingredients under different storage conditions is needed from the manufacturers.
4. The randomization of nets to village compounds, and the differing compound size, meant that an unequal number of the two net types were distributed. This, coupled with the high attrition rate, meant that there were insufficient nets for the stated sample sizes in later sampling rounds. The original sample size calculations [18] assumed a 10% loss rate every year but in reality, the attrition rate was much higher.
5. The WHO proportionate Hole Index (pHI) was used to measure the number of holes on the net. This index does not account for the location of the holes yet several studies [30, 31] have shown that host-seeking *Anopheles* mosquitoes are more active at the top of the nets compared to the sides, suggesting that holes on the top might give easy access into the nets. Consideration should be given to amending the pHI to weight holes depending on their location.
6. More succinct surveys, asking fewer questions would improve the quality of the data on survivorship and net attrition. The lengthy data on household income, level of education etc. (Additional file 3) were not needed for the stipulated analysis. Furthermore, the head of the household, typically male, was interviewed for the surveys whereas several of the questions, such as household composition, bed net usage and net washing patterns would have been more appropriately addressed by the female household head.
7. The data strongly suggests that nets sampled at the end of the study had not all been in use for the full 3 years making it difficult to draw firm conclusions about the longevity of individual nets. Furthermore, a high proportion of the nets moved between houses in the study. Amending the data collection tools to track the location of each of the distributed nets, regardless of whether they were located in the originally assigned household, would enable more precise measurements of net survivorship.

Finally, the current study involved 6 monthly assessments, enabling us to detect seasonal variations in net usage. Other durability studies typically survey the nets on an annual basis and, whilst this is probably a more pragmatic approach for future studies given the workload involved, it is important to note that the time of year at which surveys are performed could dramatically impact the proportion of nets hanging over a sleeping space due to the variation in seasonality observed. Regardless of the frequency of the surveys, comparison between studies would be facilitated by ensuring consistent methods of data collection and reporting between studies.

## Conclusion

This is the first report of a full 3-year durability study on a combination or ‘next generation’ net. Although a clinical trial of the PPF-Permethrin Olyset^®^ Duo net showed this net provides better protection from malaria than standard pyrethroid only nets, it is unlikely that, based on the results from the current study, this particular product would meet the WHO’s definition of a ‘long-lasting insecticidal net’. It is of concern that there are now many new types of nets in operational use, including PBO-pyrethroid nets and, from 2019, dual active ingredient nets, for which no data on their durability and longitudinal performance in the field is available. Furthermore, the poor performance of a conventional LLIN in this study highlights that all net types must be continuously monitored for their durability, including the ones that are currently used as standard LLINs.

## Supplementary information

**Additional file 1.** Information sheet and consent form.

**Additional file 2.** Study profile.

**Additional file 3.** Questionnaire administered to heads of households.

**Additional file 4.** Adjusted mortality of susceptible *An. gambiae* (Kisumu strain) mosquitoes exposed in cone bioassays to PPF-permethrin nets and LLINs at LSTM.

**Additional file 5.** Adjusted knockdown of susceptible *An. gambiae* (Kisumu strain) mosquitoes exposed in cone bioassays to PPF-permethrin nets and LLINs.

**Additional file 6.** Adjusted knockdown of resistant *An. gambiae* (Tiassalé strain) mosquitoes exposed in cone bioassays to LLINs and PPF-permethrin nets at LSTM.

**Additional file 7.** Fecundity and fertility study result for resistant *An. gambiae* (Tiassalé 13 strain) mosquitoes exposed in cone bioassays to PPF-permethrin nets and LLINs.

## Data Availability

The datasets used and/or analysed during the current study are available from the corresponding author on reasonable request.

## Abbreviations

CNRFP: Centre National de Recherche et de Formation sur le Paludisme
DCP: dicyclohexyl phthalate
GLMM: Generalized Linear Mixed Model
LLINs: long-lasting insecticidal nets
LRTs: log-likelihood ratio tests
LSTM: Liverpool School of Tropical Medicine
PBO: pyriproxyfen
pHI: proportionate hole index
WHO: World Health Organization
