# Correction to: Assessing the impact of the addition of pyriproxyfen on the durability of permethrin-treated bed nets in Burkina Faso: a compound-randomized controlled trial

**DOI:** 10.1186/s12936-020-03429-9

**Published:** 2020-10-06

**Authors:** Kobié H. Toé, Frank Mechan, Julie-Anne A. Tangena, Marion Morris, Joanna Solino, Emile F. S. Tchicaya, Alphonse Traoré, Hanafy Ismail, James Maas, Natalie Lissenden, Margaret Pinder, Steve W. Lindsay, Alfred B. Tiono, Hilary Ranson, N’Falé Sagnon

**Affiliations:** 1 Centre National de Recherche et de Formation sur le Paludisme, Ouagadou-gou, Burkina Faso; 2 Vector Biology Department, Liverpool School of Tropical Medicine, Liverpool, UK; 3 Swiss Centre for Scientifc Research in Côte d’Ivoire, Abidjan, Côte d’Ivoire; 4 Department of Biosciences, Durham University, Durham, UK; 5 Medical Research Council Unit, The Gambia at the London School of Hygiene and Tropical Medicine, Banjul, The Gambia

## Correction to: Malar J 18:383 (2019) 10.1186/s12936-019-3018-1

Following publication of the original article [1], the authors reported an error in one of the coordinates provided in the subsection ‘Study design and study area’.

Where it says “10° 31′ 6.07″ N, 4° 18′ 48.01″ E”, it should instead say “10° 31′ 6.07″ N, 4° 18′ 48.01″ W” (that is, West instead of East).

Similarly, where is says “10° 36′ 17.56″ N, 4° 22′ 22.17″ E”, it should instead say “10° 36′ 17.56″ N, 4° 22′ 22.17″ W” (that is, West instead of East).

The authors apologize for this error.
