# Supplementary material for: Assessing the impact of the addition of pyriproxyfen on the durability of permethrin-treated bed nets in Burkina Faso: a compound-randomized controlled trial
Source: Malar J. 2019 Dec 2;18:383. doi: 10.1186/s12936-019-3018-1 (PMC6889366; doi:10.1186/s12936-019-3018-1)
Supplement: Supplementary file 1 — Additional file 1. Information sheet and consent form. [file 12936_2019_3018_MOESM1_ESM.pdf]

Supplementary S1 Information sheet and consent form

PARTICIPANT INFORMATION AND INFORMED CONSENT AGREEMENT FORM FOR SELECTED HOUSEHOLDS

Version 2.0 – 13rd May 2014

To assess whether addition of pyriproxyfen to long-lasting insecticidal mosquito nets increases their durability compared to standard long-lasting insecticidal mosquito nets. Protocol for a cluster randomized study.

Document type: INFORMATION SHEET AND INFORMED CONSENT FORM

Based on: Protocol, v2.0, dated 13rd – May - 2014

Participant ID ________________________

Introduction

My name is <………………..>, and I have with me <………………..> and I / we work for Centre National de Recherches et Formation sur la Paludisme. I invite you to read carefully this document / or to listen carefully to as we explain it to you in a language you understand well, before accepting to participate in this study. The aim of this study is to check the action of a new type of mosquito net treated with insecticide. We expect that the mosquito nets will retain their power to kill malaria mosquitoes after several washes and 3 or more years of use.

This study has been cleared by the ethical committee of Burkina Faso.

Purpose and Background of the study

First some background information. As you probably know, malaria is a major disease in Burkina Faso and is transmitted from one person to another through the bites of certain mosquitoes. These mosquitoes usually bite after dark. Sleeping under a mosquito net protects against mosquitoes that bite in the night. If the net has been treated with a chemical that kills insects (insecticides), it gives better protection against mosquito bites. Some kinds of nets are given a special treatment in the factory and do not require retreatment until the end of their useful life; these are called long-lasting insecticidal mosquito nets (LLINs).

We are distributing two types of LLIN for malaria prevention and control in areas targeted for this intervention. The community you live in has been targeted to receive both types of nets. We want to measure how long each type of net actually last in routine use in the households in your community. Your village has been selected for this study. As you may be aware, we first asked your community leaders to give permission for this study. Then, the nets were distributed, such that every household has at least one study net to sleep under. My team has now come to your village, in consultation with your community leader or village headperson, to find out the number of months that these nets are used over beds /sleeping place and how long the chemical lasts.

Information on study nets

The study nets we will give to your household are factory-treated nets. In this study, the chemicals used are: permethrin alone or permethrin and pyriproxyfen combined. Note that these products are not new: they are all well-established, but they have not been used together on a net before. We are currently conducting a study in other villages near here to measure whether the nets with the two chemicals are better at reducing malaria than those with the one chemical. The question we are asking in this study is "how long do these nets last?".

Type of study

We will provide your household with new nets, some of each type, and ask you to hang them over your beds / sleeping places. We will take away the nets you have at the moment to avoid confusion. We will then follow the nets over time, to see how quickly they get holes and wear out and how much chemical they still have, by choosing a few nets to collect data on in 6 months time, then after 12,18,24, 30 and 30 36 months. The choice of nets to collect data on will be by lottery. If we take your net for measuring the chemical then you will be given a new net. A smaller number of these households, chosen at random, are being re-visited every few months, to see whether the nets are still in use and still in good condition.

Participant selection
After this area was selected for the study, we gave each household a number. In order to pick households for the follow-up visits, we used a computer to choose numbers like in a lottery, and that is how we chose the nets to check. We are asking you for an interview because your net is one of the selected nets, or because you are the <parent / guardian / head of household> of a child who uses one of the selected nets.

Procedures
I would therefore like to have your consent to be interviewed; this will last about <approximate time > minutes. During the interview, I will ask you some questions about your household, the status of the net given to you or your child and how you use and handle your net. I will ask you to show the net to me in your room if you are agree, so I can see how much it has worn.

<NOTE to interviewer: If the net has been selected for bioassay testing, read 'A' below; alternatively, if this net has not been selected for bioassay testing, then read 'B'.>

A – We would like to take some nets away with us, and measure how much of the insecticide is still there and how much has worn off. Your net has been selected at random for this testing. If you agree to give us permission to take away this net, then I will immediately give you a replacement net, which will be yours to keep and you can put it on the bed today.

B – We will not damage the net, and after the interview, we will return it. At the completion of the study, all villagers will be told the main outcomes of the study in a community meeting in the village.

Confidentiality
All information related to your participation will be kept confidential and will not be revealed to anyone, except if required by law, such as in a legal request for the list of beneficiaries. Your identity will not be revealed in any reports or publications resulting from the study. The results of the interview will be put into a computer, but with the code numbers of the household, and without the names of the people interviewed. The data collected will be kept for analysis. It will be stored for some time on paper and in the computer, but may eventually be destroyed.

Voluntary participation: right to refuse or withdraw consent
Your participation in the interview is entirely voluntary. You are not under any obligation to participate and you have the right to refuse this invitation. If at any time during the interview, you decide not to participate further, you are free to withdraw immediately, with no further discussion; this will have no adverse consequences for you. Whether you choose to participate or not, you will still receive all the public services you usually do. The study nets that have been given to your household belong to you and are yours to keep. In a few cases, we may ask you to give an old net back to us in exchange for a replacement new one, but you may refuse this request if you wish.

Who to contact

If you have any questions, please ask them, either now or later. If you wish to ask questions later, you may contact any of the following:

[redacted]

Any important new information concerning the results of our study will be made known to you.

This proposal has been reviewed and approved by ethics committee of Burkina Faso, whose task it is to make sure that study participants' right is respected If you wish to find out more about these committees, please contact :

EC Chairman: [redacted]

IRB (CIB/CNRFP) Chairman: [redacted]

We are leaving a copy of this informed consent form with you for your information and future

reference.

INFORMED CONSENT FORM

I have read this information in French or it has been read to me in my native language. I have had the opportunity to ask questions about it, and any questions that I have asked have been answered to my satisfaction. I consent voluntarily to participate in this study, and I understand that I have the right to withdraw from the study at any time without in any way affecting my rights. I also understand that the principal investigator of the study can exclude my household from the study without my consent. I have been given a copy of this consent form.

Print name of participant:____

Date and signature of participant

____________________________________ __/___/___ (dd/mm/yy)

If illiterate

I have witnessed the interviewer reading the consent form to the potential participant. The reading was careful and accurate and the individual had the opportunity to ask questions. I confirm that the individual has given consent freely.

Print name of independent literate witness:__________________________________

(If possible, this person will be selected by the participant and will have no connection to the research team.)

Signature of the witness and date

_______________________________________ ___/___/___ (dd/mm/yy)

I have read or witnessed the reading of the consent form to the potential participant. The reading was careful and accurate and the individual had the opportunity to ask questions. I confirm that the individual has given consent freely.

Print name of researcher

Date and signature of researcher

____ ___/___/___ (dd/mm/yy)
