# Supplementary figures and images for: Assessing the impact of the addition of pyriproxyfen on the durability of permethrin-treated bed nets in Burkina Faso: a compound-randomized controlled trial
Source: Malar J. 2019 Dec 2;18:383. doi: 10.1186/s12936-019-3018-1 (PMC6889366; doi:10.1186/s12936-019-3018-1)

# Figure S2 Study profile


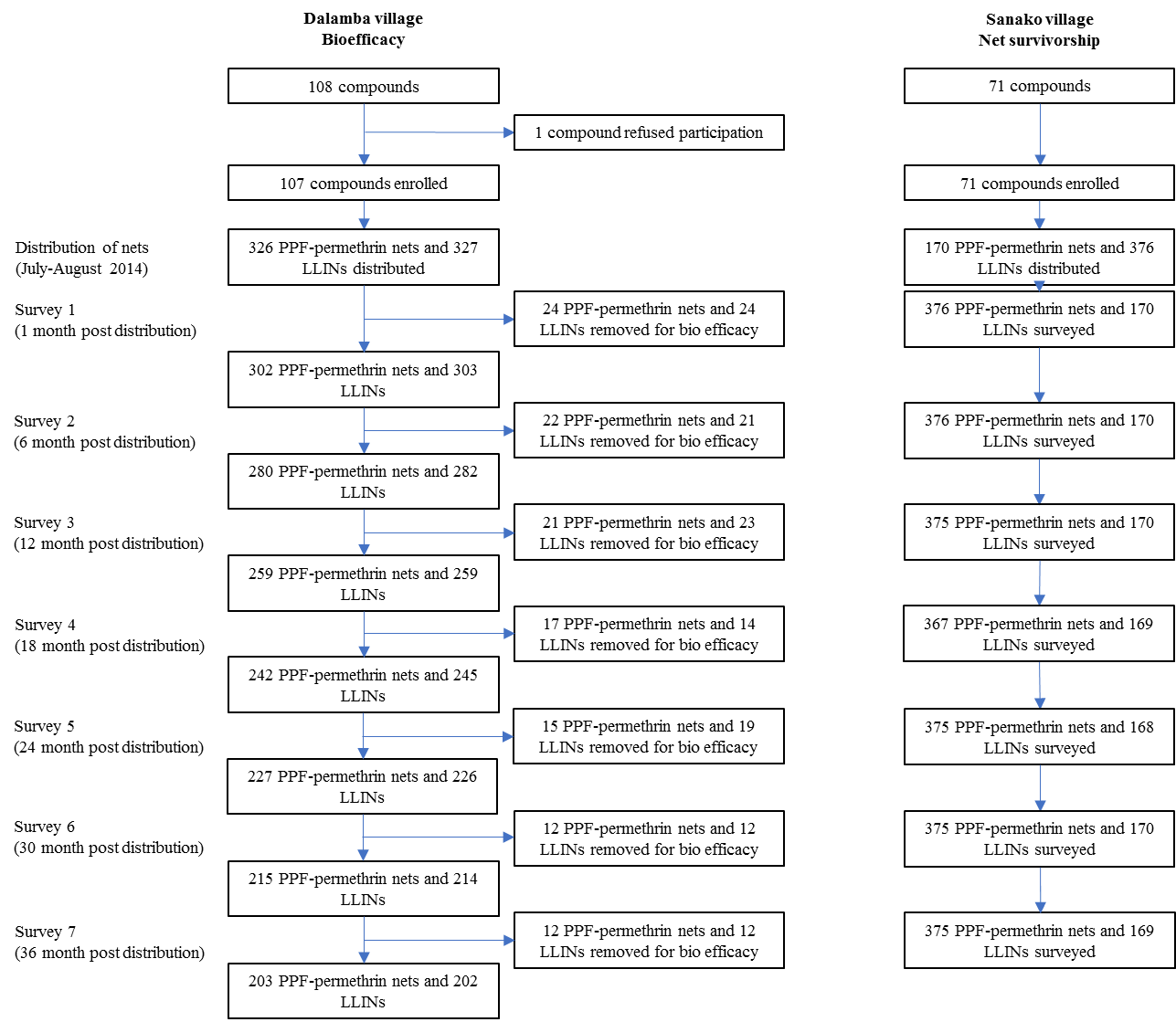

Supplement: Supplementary file 2 — Additional file 2. Study profile. [file 12936_2019_3018_MOESM2_ESM.docx]
