# Supplementary material for: Assessing the impact of the addition of pyriproxyfen on the durability of permethrin-treated bed nets in Burkina Faso: a compound-randomized controlled trial
Source: Malar J. 2019 Dec 2;18:383. doi: 10.1186/s12936-019-3018-1 (PMC6889366; doi:10.1186/s12936-019-3018-1)
Supplement: Supplementary file 3 — Additional file 3. Questionnaire administered to heads of households. [file 12936_2019_3018_MOESM3_ESM.docx]

# Additional S3 Questionnaire

QUESTIONNAIRE FOR MONITORING DURABILITY OF NETS UNDER OPERATIONAL CONDITIONS

Number of survey I____I

LLIN Identification number I____I____I____I **(to be filled in by supervisor)**

0.1 Code of interviewer I____I____I

0.2 Date Day / month / year I___I___I / I___I___I / I___I___I___I___I

0.3 Name of village I________________________________________________I

0.4 Compound identification number I____I____I____I_____I

0.5 Compound identification number I____I____I____I____I

**To be filled in by the supervisor at the end of the day**

0.6 Code of supervisor I____I____I

Comments________________________________________________________________________________________________________________________

I confirm that the questionnaire is complete.

Date: I____I____I / I____I____I / I____I____I____I____I

Name: I_________________________________________________________I

Signature I______________________________________________________________I

**To be filled in by data entry clerks during data entry**

| Data entry clerk 1 | Data entry clerk 2 |
| --- | --- |
| Date  I___I___I / I___I___I / I___I___I___I___I | Date  I___I___I / I___I___I / I___I___I___I___I |
| Signature______________________ | Signature______________________ |

| **Section 1: *I would like to ask you (head of household or adult > 18 years) some questions about your household*** | | | | | |
| --- | --- | --- | --- | --- | --- |
| **Q#** | | | **Questions & filters** | **Coding category** | **Answer (enter coding category** |
| 1.1 | | | Who is responding to the questions? | 1….Yes  0….No | Parent or guardian of user(s) of net I____I  Other adult in household I____I |
| 1.2 | | | What is the highest level of education of the head of the household? | 1….None  2….Religious school  3….Primary school  4....Secondary school  5….Higher education  6….Other, specify | I____I  Other ________________________ |
| 1.3 | | | Does your household have electricity? | 1….Yes  0….No | I____I |
| 1.4 | | | What is the principal type of toilet facility used by members of the household? | 1….Own flush toilet  2….Shared flush toilet  3….Own pit latrine  4….Shared pit latrine  5….Bush or field  6….Other | I____I  Other  _____________________ |
| 1.5 | | | What is the principal household source of drinking-water? | 1….Piped water into home  2….Protected well in home  3….Unprotected well in yard  4….Open well in yard  5….Protected well in yard  6….Unprotected public well  7….Protected public well  8….Tap in yard  9….Tanker truck  10...Bottled water  11...Public tap  12…Rainwater  13…Surface water  14…Spring  15…Other | I____I  Other  _____________________ |
| 1.6 | | | How many people slept in your household last night? |  | Adults > 15 years I____I____I  5–15 years I____I____I  < 5 years I____I____I |
| 1.7 | | | How many sleeping places were used last night in your household?  *(including sleeping places outside and temporary spaces)* |  | \|____\|____I |
| 1.8 | | | How many mosquito nets that can be used for sleeping does your household have?  *(Probe for any nets currently not in use: stored, saved, still in packaging)* |  | \|____\|____I |
| Section 2: Net use and handling | | | | | |
| 2.1 | | Has this net ever been used for sleeping under? | | Yes  No -> End questionnaire | I____I |
| 2.2 | | Was this net used last night to sleep under? | | 1….Yes -> Skip to 3.4  0….No | I____I |
| 2.3 | | If No, why was this net not used last night? | | 1….Yes  0….No | Too hot I____I  Don’t like the smell I____I  Feel “closed in” I____I  No malaria now I____I  No mosquitoes I____I  The net is too torn or old I____I  Net not available I____I  Used another net I____I  User did not sleep here I____I  Other I____I______________  Don’t know I____I |
| 2.4 | | In the past week, how often was the net used? | | 1….Every night (7 nights)  2….Most nights (5–6 nights)  3….Some nights (1–4)  4….Not used at all (0 nights)  9….Don’t know | I____I |
| 2.5 | | How many adults (> 15 years) slept under this net last night? | |  | I____I |
| 2.6 | | How many children 5–15 years slept under this net last night? | |  | I____I |
| 2.7 | | How many children < 5 years slept under this net last night? | |  | I____I |
| 2.8 | | During which periods of the year is this net used to sleep under? | | 1… All year  2….Only the rainy season  3….Only the dry season  9….Don’t know | I____I |
| 2.9 | | Is this net ever used for sleeping under away from the main house? If yes, where? | | 1….Taken to the fields  2….Taken to the beach  3….Taken to the forest  4….Taken to the farm hut  5….Other, specify  6….Not used away ->Skip to  Q.3.11  9….Don’t know ->Skip to Q. 3.11 | I____I  Other  _________________________ |
| 2.10 | | During which periods of the year is this net used to sleep under away from the main house? | | 1… All year  2….Only the rainy seasons  3….Only the dry season  9….Don’t know | I____I |
| 2.11 | | Has this net ever been used over the following types of sleeping places? | | 1…Yes  0…No | Reed mat I____I  Cut bamboo I____I  Grass I____I  Foam mattress I____I  Wooden bed frame (finished) I____I  Wooden bed frame (sticks) I____I  Metal bed frame I____I  Bare floor or ground I____I  Other, specify I____I  ________________________ |
| 2.12 | | Do you tuck the net in at night? | | 1….Yes  0….No  9….Don’t know | I____I |
| 2.13 | | Has the net ever been washed? | | 1….Yes  0….No ->Skip to Q.4.1  9….Don’t know ->Skip to Q.4.1 | I____I |
| 2.14 | | When was the last time you washed the net? | | 1…1 week ago  2….1 week to 1 month ago  3….1–3 months ago  4….3–6 months ago  5….> 6 months ago  9….Don’t know | I____I |
| 2.15 | | What type of soap was used? | | 1….None  2….Local bar soap  3….Detergent powder  4….Mix (bar and detergent)  5….Bleach  9….Don’t know | I____I |
| 2.16 | | How long did the net soak for? | | 1….Did not soak the net  2….< 1 h  3…..> 1 h  9….Don’t know | I____I |
| 2.17 | | Was the net scrubbed hard or beaten on a hard surface (e.g. rocks, with sticks)? | | 1….Yes  0….No  9….Don’t know | I____I |
| 2.18 | | Where was the net dried? | | 1….Outside in the sun  2….Outside in the shade  3….Inside  9….Don’t know | I____I |
| Section 3: LN condition | | | | | |
| 3.1 | In the past month, have any new holes appeared in the net that you are aware of? | | | 1….Yes  0….No  9….Don’t know | I____I |
| 3.2 | What caused these new holes? | | | 1….Yes  0….No | Tore or split when caught on an object I____I  Was burned I____I  Was caused by animals I____I  Children \|____\|  In another way I_____I,  specify  Don’t know I____I |
| 3.3 | How is the net found? (Observe) | | | 1…. Hanging loose over  sleeping place  2….Hanging tied in knot  3….Hanging folded  4….Visible but not hung up  5….Stored away | I____I |
| 3.4 | What type of sleeping place is the net hanging over? (Observe) | | | 1….Reed mat  2….Cut bamboo  3….Grass  4….Foam mattress  5….Wooden bed frame  (finished)  6….Wooden bed frame  (sticks)  7….Metal bed frame  8….Nothing  9….Other, specify | I____I  Other  _________________________ |
| 3.5 | Where is it found? (Observe) | | | 1….Inside  2….Outside -> Skip to Q.4.9 | I____I |
| 3.6 | What is the principal type of flooring in the room where the net is found?  (Observe) | | | 1….Soil or sand  2….Wood, palm, bamboo  3….Cement (including vinyl)  4….Cement  5….Carpet  6….Other | I____I  Other  _____________________ |
| 3.7 | What are the walls of the room in which the net is found in made of?  (Observe) | | | 1….Mud brick  2….Mud with wood frame  2….Concrete  3….Twigs  4….Wood  5….Straw  6….Bamboo  7….Corrugated iron  8….Lime-plastered  9….No walls (used outside)  10… Other, specify | I____I____I  Other  _________________________ |
| 3.8 | What is the roof or ceiling of the room in which the net is found in made of? (Observe) | | | 1….Grass thatch  2….Corrugated iron  3….Concrete  4….Reed mats  5….Wood  6….Tiles  7….Other, specify | I____I  Other  _________________________ |
| 3.9 | Do you use an open flame for cooking, heating or lighting where the net is found? | | | 1….Yes  0….No | Wood fire I____I  Charcoal fire I____I  Wax candle I____I  Oil lamp with a glass I____I  Oil lamp without glass I____I  Other, specify I____I  _________________________ |
| 3.10 | What type of holes are observed? | | | 1….Yes  0….No | Horizontal tears at bottom I____I  Holes at hanging points I____I  Open seams I____I  Burn holes I____I  Holes from rodents I____I  Whole section missing I____I |
| 3.11 | Number of holes of size 1 Less than size of thumb (0.5–2 cm) | | |  | Roof I____I____I  Upper I____I____I  Lower I____I____I  Seams I____I____I |
| 3.12 | Number of holes of size 2 Larger than thumb, smaller  than fist (2–10 cm) | | |  | Roof I____I____I  Upper I____I____I  Lower I____I____I  Seams I____I____I |
| 3.13 | Number of holes of size 3 Larger than fist, smaller than  head (10–25 cm) | | |  | Roof I____I____I  Upper I____I____I  Lower I____I____I  Seams I____I____I |
| 3.14 | Number of holes of size 4 Larger than head (> 25 cm) | | |  | Roof I____I____I  Upper I____I____I  Lower I____I____I  Seams I____I____I |
| 3.15 | Number of holes repaired | | |  | Stitched I____I____I  Knotted I____I____I  Patched I____I____I |
