# Supplementary material for: Assessing the impact of the addition of pyriproxyfen on the durability of permethrin-treated bed nets in Burkina Faso: a compound-randomized controlled trial
Source: Malar J. 2019 Dec 2;18:383. doi: 10.1186/s12936-019-3018-1 (PMC6889366; doi:10.1186/s12936-019-3018-1)
Supplement: Supplementary file 4 — Additional file 4. Adjusted mortality of susceptible An. gambiae (Kisumu strain) mosquitoes exposed in cone bioassays to PPF-permethrin nets and LLINs at LSTM. [file 12936_2019_3018_MOESM4_ESM.docx]

**
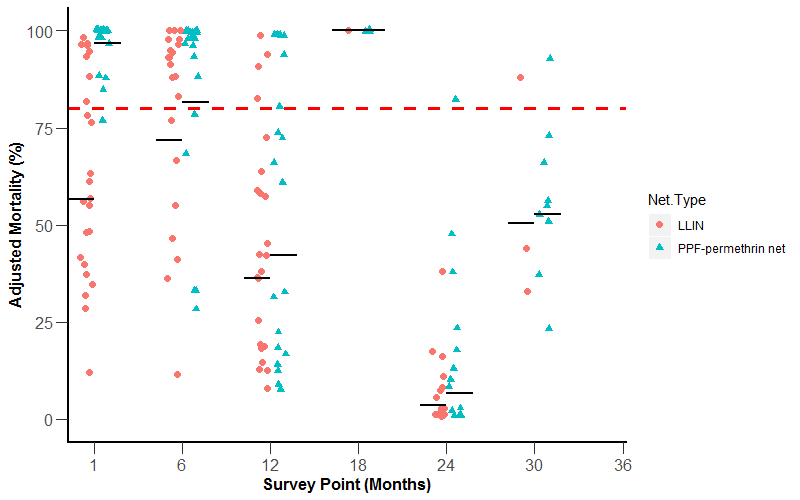
**

**Figure S4 Adjusted mortality of susceptible *An. gambiae* (Kisumu strain) mosquitoes exposed in cone bioassays to LLINs and PPF-permethrin nets at LSTM.** Horizontal black bars indicate geometric mean mortality. Horizontal red dotted line indicates 80% mortality threshold. . • Standard permethrin-treated long-lasting insecticide treated net and ▲Pyriproxyfen-permethrin treated net
