# Supplementary material for: Assessing the impact of the addition of pyriproxyfen on the durability of permethrin-treated bed nets in Burkina Faso: a compound-randomized controlled trial
Source: Malar J. 2019 Dec 2;18:383. doi: 10.1186/s12936-019-3018-1 (PMC6889366; doi:10.1186/s12936-019-3018-1)
Supplement: Supplementary file 5 — Additional file 5. Adjusted knockdown of susceptible An. gambiae (Kisumu strain) mosquitoes exposed in cone bioassays to PPF-permethrin nets and LLINs. [file 12936_2019_3018_MOESM5_ESM.docx]

| A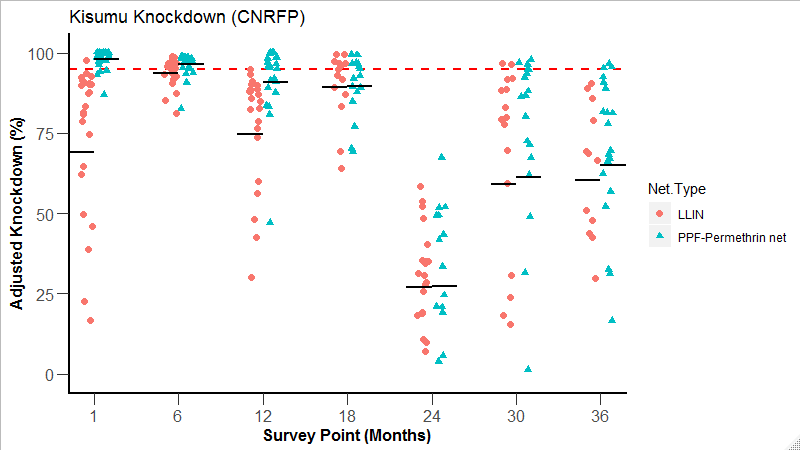 | 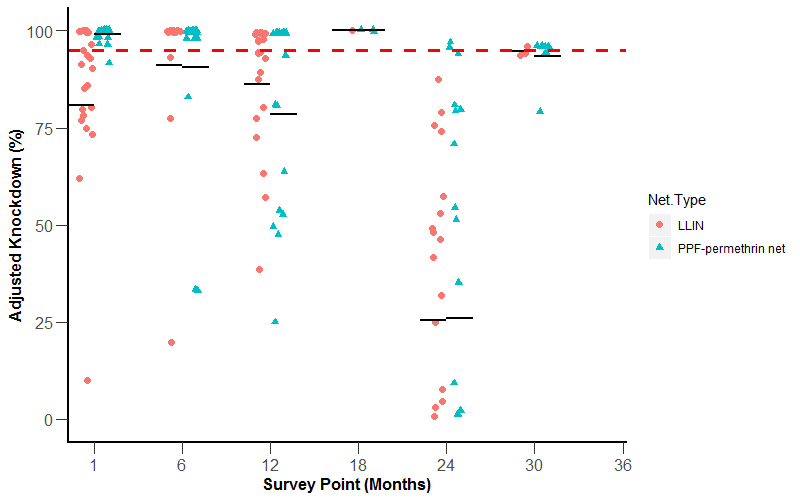B |
| --- | --- |
| Figure S5 Adjusted knockdown of susceptible *An. gambiae* (Kisumu strain) mosquitoes exposed in cone bioassays to PPF-permethrin nets and LLINs (A) at CNRFP (B) at LSTM. • Standard permethrin-treated long-lasting insecticide treated net and ▲Pyriproxyfen-permethrin treated net. Horizontal red dotted line indicates 95% knockdown threshold. | |
