# Supplementary material for: Assessing the impact of the addition of pyriproxyfen on the durability of permethrin-treated bed nets in Burkina Faso: a compound-randomized controlled trial
Source: Malar J. 2019 Dec 2;18:383. doi: 10.1186/s12936-019-3018-1 (PMC6889366; doi:10.1186/s12936-019-3018-1)
Supplement: Supplementary file 6 — Additional file 6. Adjusted knockdown of resistant An. gambiae (Tiassalé strain) mosquitoes exposed in cone bioassays to LLINs and PPF-permethrin nets at LSTM. [file 12936_2019_3018_MOESM6_ESM.docx]

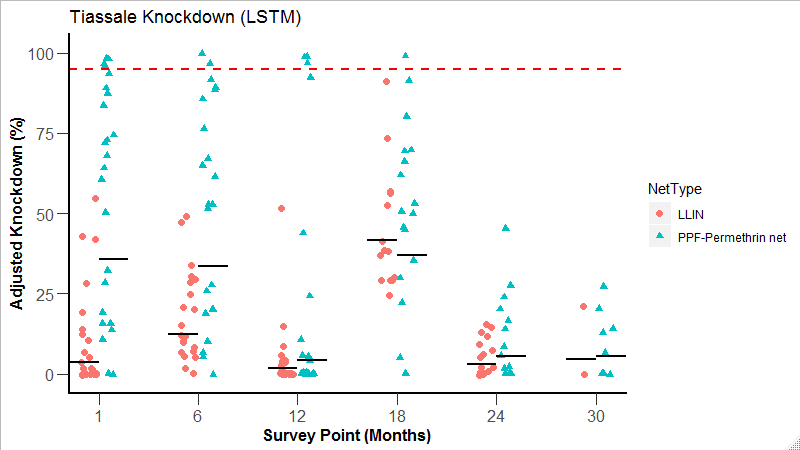


# Figure S6 Adjusted knockdown of resistant *An. Gambiae*. (Tiassalé strain) mosquitoes exposed in cone bioassays to LLINs and PPF-permethrin nets at LSTM. Horizontal red dotted line indicates 95% knockdown threshold.
