# Supplementary material for: Assessing the impact of the addition of pyriproxyfen on the durability of permethrin-treated bed nets in Burkina Faso: a compound-randomized controlled trial
Source: Malar J. 2019 Dec 2;18:383. doi: 10.1186/s12936-019-3018-1 (PMC6889366; doi:10.1186/s12936-019-3018-1)
Supplement: Supplementary file 7 — Additional file 7. Fecundity and fertility study result for resistant An. gambiae (Tiassalé 13 strain) mosquitoes exposed in cone bioassays to PPF-permethrin nets and LLINs. [file 12936_2019_3018_MOESM7_ESM.docx]

# Table S7 Fecundity and fertility study result for resistant *An. gambiae* s.s. (Tiassalé 13 strain) mosquitoes exposed in cone bioassays to PPF-permethrin nets and LLINs

|  | **1 month** | | | **6 months** | | | **12 months** | | | **18 months** | | | **24 months** | | |
| --- | --- | --- | --- | --- | --- | --- | --- | --- | --- | --- | --- | --- | --- | --- | --- |
|  | **C** | **LLIN** | **PPF-permethrin** | **C** | **LLIN** | **PPF-permethrin** | **C** | **LLIN** | **PPF-permethrin** | **C** | **LLIN** | **PPF-permethrin** | **C** | **LLIN** |  |
| **BF and survived** | 126 | 506 | 318 | 82 | 609 | 450 | 120 | 908 | 609 | 42 | 355 | 317 | 218 | 773 | 645 |
| **oviposition rate (N)** | 0.55  (69) | 0.58  (295) | 0.33*  (104) | 0.96  (79) | 0.42*  (258) | 0.45*  (201) | 0.46  (55) | 0.50  (451) | 0.52  (315) | 0.43  (18) | 0.51  (180) | 0.49  (155) | 0.45  (98) | 0.50  (383) | 0.55  (357) |
| **Fecundity (95% CI)** | 36.4  (23.8-49.0) | 44.8  (36.6-53.0) | 19.4*  (12.3-26.6) | 25.5  (12.9-38.0) | 23.6  (18.8-28.4) | 23.8  (18.3-29.3) | 33.4  (20.9-45.8) | 35.2  (30.2-40.2) | 32.6  (24.6-40.6) | 31.8 | 34.5  (26.3-42.7) | 24.9  (18.2-31.7) | 29.1  (20.9-37.3) | 34.7  (25.5-44.0) | 34.6  (29.6-39.6) |
| **Eggs per mosquito that laid eggs** | 57.2  (46.3-68.1) | 62.5  (58.7-66.4) | 57.9  (42.0-73.9) | 30.4  (18.6-42.1) | 57.2*  (50.0-64.3) | 55.2*  (45.6-64.7) | 67.0  (57.3-76.6) | 68.8  (64.1-73.4) | 65.8  (57.9-73.7) | 74.2 | 65.2  (58.2-72.2) | 50.1  (40.7-59.4) | 67.4  (59.3-75.4) | 67.6  (59.7-75.6) | 63.4  (56.5-70.2) |
| **Hatch rate (95% CI)** | 0.43  (0.32-0.53) | 0.43  (0.39-0.47) | 0.17*  (0.10-0.24) | 0.47  (0.32-0.62) | 0.37  0.32-0.43) | 0.31*  (0.24-0.39) | 0.59  (0.49-0.70) | 0.52  (0.50-0.55) | 0.47*  (0.41-0.54) | 0.27 | 0.43  (0.34-0.52) | 0.33  (0.28-0.38) | 0.65  (0.53-0.78) | 0.75  (0.69-0.81) | 0.70  (0.62-0.78) |
| **Fertility**  **(95% CI)** | 17.2  (9.2-25.2) | 19.7  (15.3-24.1) | 3.6*  (1.7-5.4) | 11.5  (5.5-17.6) | 9.0  (6.6-11.4) | 8.4  (5.7-11.2) | 20.2  (11.5-28.9) | 18.5  (15.5-21.4) | 15.8  (11.5-20.0) | 8.4 | 15.4  (10.0-20.8) | 7.8  (4.8-10.8) | 20.2  (13.4-27.1) | 26.0  (18.6-33.4) | 24.4  (19.6-29.3) |

C: control, LLIN: permethrin treated nets, PPF-LLIN: Pyriproxyfen and permethrin treated nets, BF: Blood fed, N: number of mosquitoes, Oviposition rate: proportion of survived blood-fed mosquitoes that laid eggs, fecundity: number of eggs laid per survived blood fed female, hatch rate: proportion of eggs that hatched, fertility: number of hatched eggs per survived blood fed female, 95% CI: 95% confidence interval. * represents significant difference compared to the control netting.
